# Single-Atom Colloidal Nanorobotics Enhanced Stem Cell Therapy for Corneal Injury Repair

**DOI:** 10.1021/acsnano.4c18874

**Published:** 2025-05-13

**Authors:** Xiaohui Ju, Eliška Javorková, Jan Michalička, Martin Pumera

**Affiliations:** 1 Future Energy and Innovation Laboratory, Central European Institute of Technology, 48274 Brno University of Technology, Purkyňova 123, Brno 61200, Czech Republic; 2 Department of Toxicology and Molecular Epidemiology, Institute of Experimental Medicine, 48311 Academy of Sciences of the Czech Republic, Vídeňská 1083, Prague 14200, Czech Republic; 3 Department of Cell Biology, Faculty of Science, Charles University, Viničná 7, Prague 12844, Czech Republic; 4 Central European Institute of Technology, 48274 Brno University of Technology, Purkyňova 123, Brno 61200, Czech Republic; 5 Advanced Nanorobots & Multiscale Robotics Laboratory, Faculty of Electrical Engineering and Computer Science, VSB-Technical University of Ostrava, 17. listopadu 2172/15, Ostrava 70800, Czech Republic; 6 Department of Medical Research, China Medical University Hospital, China Medical University, No. 91 Hsueh-Shih Road, Taichung 40402, Taiwan

**Keywords:** single-atom, cerium oxide, nanorobot, glucose, mesenchymal stem cells, cornea

## Abstract

Corneal repair using mesenchymal stem cell therapy faces challenges due to long-term cell survival issues. Here, we design cerium oxide with gold single-atom-based nanorobots (CeSAN-bots) for treating corneal damage in a synergistic combination with stem cells. Powered by glucose, CeSAN-bots exhibit enhanced diffusion and active motion due to the cascade reaction catalyzed by gold and cerium oxide. CeSAN-bots demonstrate a two-fold increase in cellular uptake efficiency into mesenchymal stem cells compared to passive uptake. CeSAN-bots possess intrinsic antioxidant and immunomodulatory properties, promoting corneal regeneration. Validation in a mouse corneal alkali burn model reveals an improvement in corneal clarity restoration when stem cells are incorporated with CeSAN-bots. This work presents a strategy for developing glucose-driven, enzyme-free, single-atom-based ultrasmall nanorobots with promising applications in targeted intracellular delivery in diverse biological environments.

## Graphical abstract

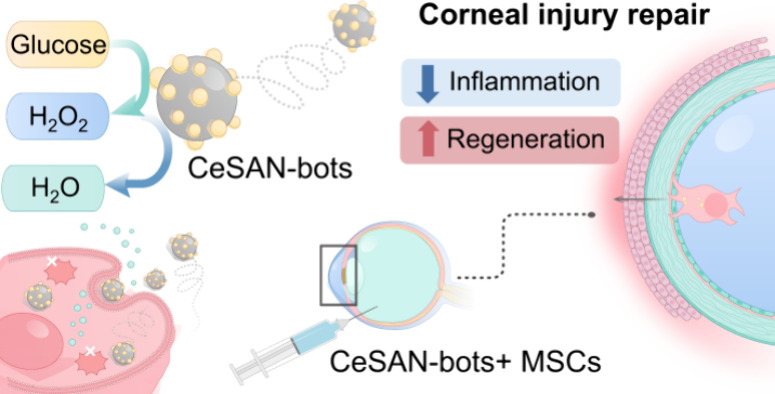

## Introduction

The cornea, possessing precise curvature for clear vision, is highly susceptible to injuries and infections due to its exposed position, leading to potential vision loss. Cornea diseases are one of the leading causes of blindness worldwide.^1^ Although clinical corneal transplantation is effective, this practice faces challenges due to graft rejection and limited availability (1/70 available).^2^ Mesenchymal stem cell (MSC)-based therapy represents a promising approach for treating ocular surface injuries in its preclinical development. MSCs promote corneal wound healing by replacing damaged cells, modulating the local environment, and creating conditions that favor regeneration and reduce inflammation. However, the low level of long-term survival rate of MSCs remains a concern due to severe oxidative stress after their migration to the injury site.^3^ Therefore, developing strategies to enhance stem cell survival under inflammatory conditions is crucial for its use in corneal regeneration.

Chemically powered micro/nanorobots hold great promise as intelligent platforms for precise targeted drug delivery in biomedical settings. Autonomous movement enables the micro/nanorobots to navigate within complex biological environments^4^ and perform encoded tasks.^5^ Among the designs, enzyme-powered micro/nanomotors can harness the catalytic power of enzymes that convert chemical fuels to motion.^6^ For glucose-driven micro/nanoswimmers, self-diffusiophoresis is generated by a localized chemical gradient formed by the asymmetrical distribution of reaction products produced by glucose oxidase (GOx).^7^ However, GOx incorporation often results in compromised catalytic activity due to disrupted enzyme structure or limited exposure of the active sites,^8^ which constrains their efficient glucose conversion and propulsion. More importantly, enzyme-powered micro/nanomotors face inherent restrictions due to protease degradation and passivation by protein corona formation.^9^

One possible approach to addressing the vulnerability of enzyme-powered micro/nanorobots is to replace enzymes with catalytic nanomaterials. Recent advances in artificial enzymes, known as nanozymes, have demonstrated remarkable potential as compared to natural enzymes.^10^ Inorganic material-based nanozymes offer solutions to challenges such as enzyme fragility, high cost, and limited reusability. In particular, nanostructured gold with GOx-like activity,^11^ which is highly effective in converting glucose to H_2_O_2_,^15^ attracts substantial interest in sensing and therapeutics. Cerium oxide nanoparticle (CeNP)-based nanozymes offer multiple enzyme-mimicking activities, such as catalase, peroxidase, superoxide dismutase, and oxidase. Their exceptional stability and minimal cytotoxicity make them a promising therapeutic agent for the treatment of damaged ocular injuries.^12^ The development of single-atom catalysts, characterized by atomically dispersed active sites, has bridged the material gap between natural enzymes and nanozymes.^13^ Downsizing functional nanoparticles to single atoms not only enhances catalytic activities and metal utilization efficiency but also facilitates the possibility of coupling cascade reactions in one confinement with structural simplicity.^14^ CeNPs supporting single-atom elements (noble metals such as Pt, Pd, and Au) with synergistic catalytic effects find their application in industrial catalytic reactions,^15^ while being less explored in biomedicine.^16^

Here, we report the design of self-powered, enzyme-free, cerium oxide-based gold single-atom-decorated nanorobots (CeSAN-bots) that display glucose-mediated propulsion and intrinsic antioxidant and immunomodulatory properties, integrated with mesenchymal stem cells for the repair of corneal injury (Figure 1). Glucose, as one of the most important nutrients with a consequent high flow from extracellular to the intracellular environment,^29^ improves the cellular uptake of CeSAN-bots in stem cells due to their enhanced diffusion and active motion. The antioxidant and immunomodulatory effect of CeSAN-bots makes MSCs more resistant to oxidative damage. *In vivo* models of mice with an alkali-burned cornea validate the synergistic effect of MSCs and CeSAN-bots promoting corneal healing. These findings broaden the scope of chemically powered nanorobots from natural enzymes to single-atom-based nanozymes, paving the way for ultrasmall, multitasking nanomachines with immense potential in precision biomedicine.

**Figure 1:**
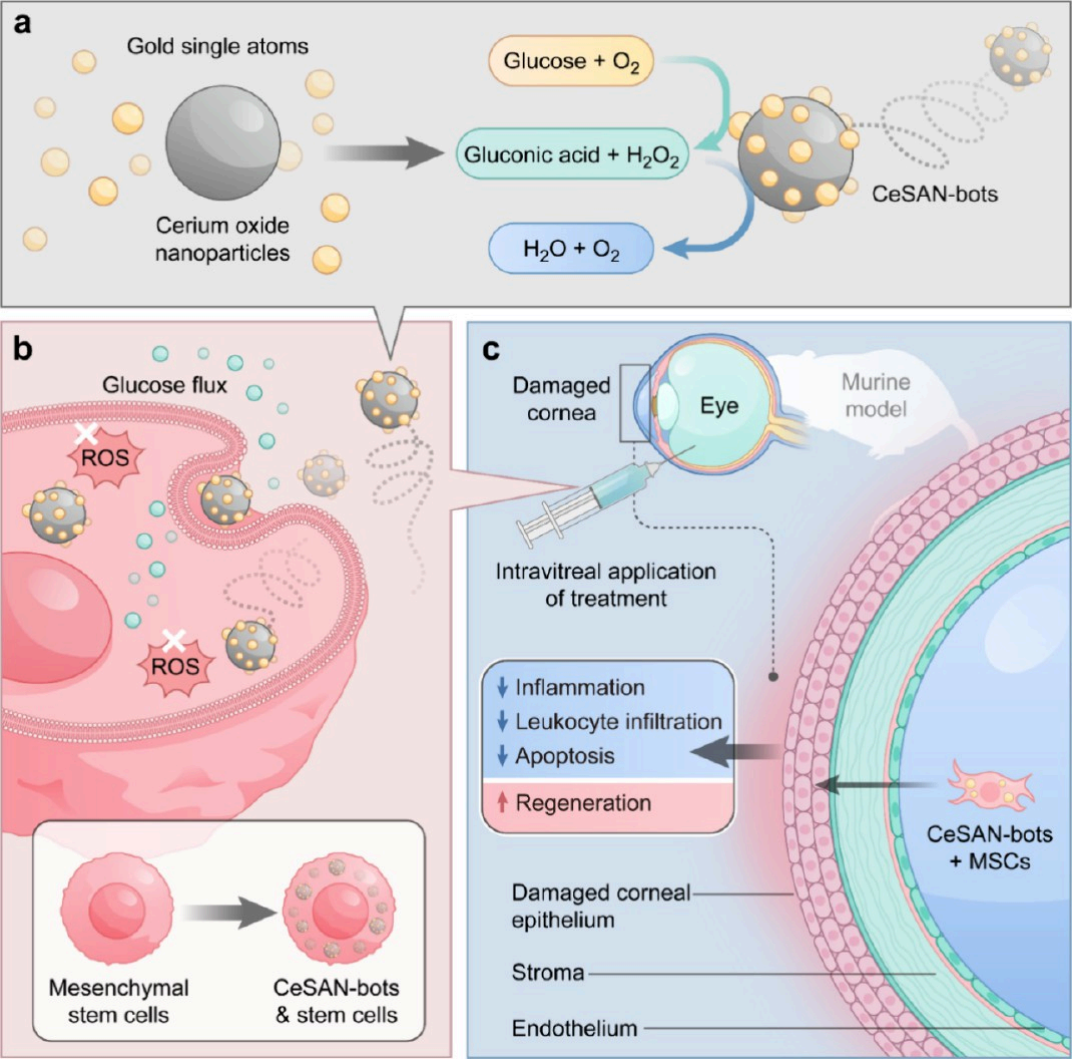
Scheme of CeSAN-bot-incorporated mesenchymal stem cells (MSCs) for corneal injury repair. (a) CeSAN-bots are assembled by decorating gold single atoms onto cerium oxide nanoparticles, which are functionalized by poly­(acrylic acid) to enhance colloidal stability and stealth in the biological environment. Assembled CeSAN-bots utilize glucose and generate a self-propelled motion. (b) Enhanced cellular uptake of CeSAN-bots by MSCs was due to enhanced diffusion. (c) Intravitreal injection of CeSAN-bot-incorporated MSCs for corneal injury repair in a mouse model.

## Results and Discussion

### Synthesis and Characterization of CeSAN-bots

The CeSAN-bots were constructed based on our previous method^17^ to synthesize colloidal poly­(acrylic acid) (PAA)-coated CeNPs (Figure 2a and Note S1). To simplify nomenclature, individual samples are named based on their metal content using the format M-CeNPs, where M represents the specific metal. When referring to all synthesized nanoparticles collectively, the term CeSAN-bots (cerium oxide-based, single-atom-decorated nanorobots) is used. Au, Pt, Ag, and Pd precursors are selected due to their reported glucose oxidation catalytic activities.^18^ Metal elements were anchored to CeNPs surfaces by single-atom noble metal deposition.^19^ The metal/Ce percentage ranges from 0.4 to 1.5 at. % (atomic percentage by X-ray photoelectron spectroscopy (XPS)). Crystallite sizes of all CeNPs show only fluorite CeO_2_ structures of 2.3 to 2.7 nm by X-ray diffraction (XRD), where no visible noble metal crystallite structures are observed. PAA provides electrostatic and steric repulsion of colloidal NP dispersions, as indicated by the negative zeta potential and confirmed by Fourier transform infrared (FTIR) spectra (Figure S1). High-resolution high-angle annular dark-field scanning transmission electron microscopy (HAADF-STEM) images show well-separated CeNPs of all four types after dispersion in a solution and drop-casting on a TEM grid coated with a carbon membrane, where the mean crystallite size of ∼3 nm could be determined for each CeNPs type (Figure S2).

**Figure 2:**
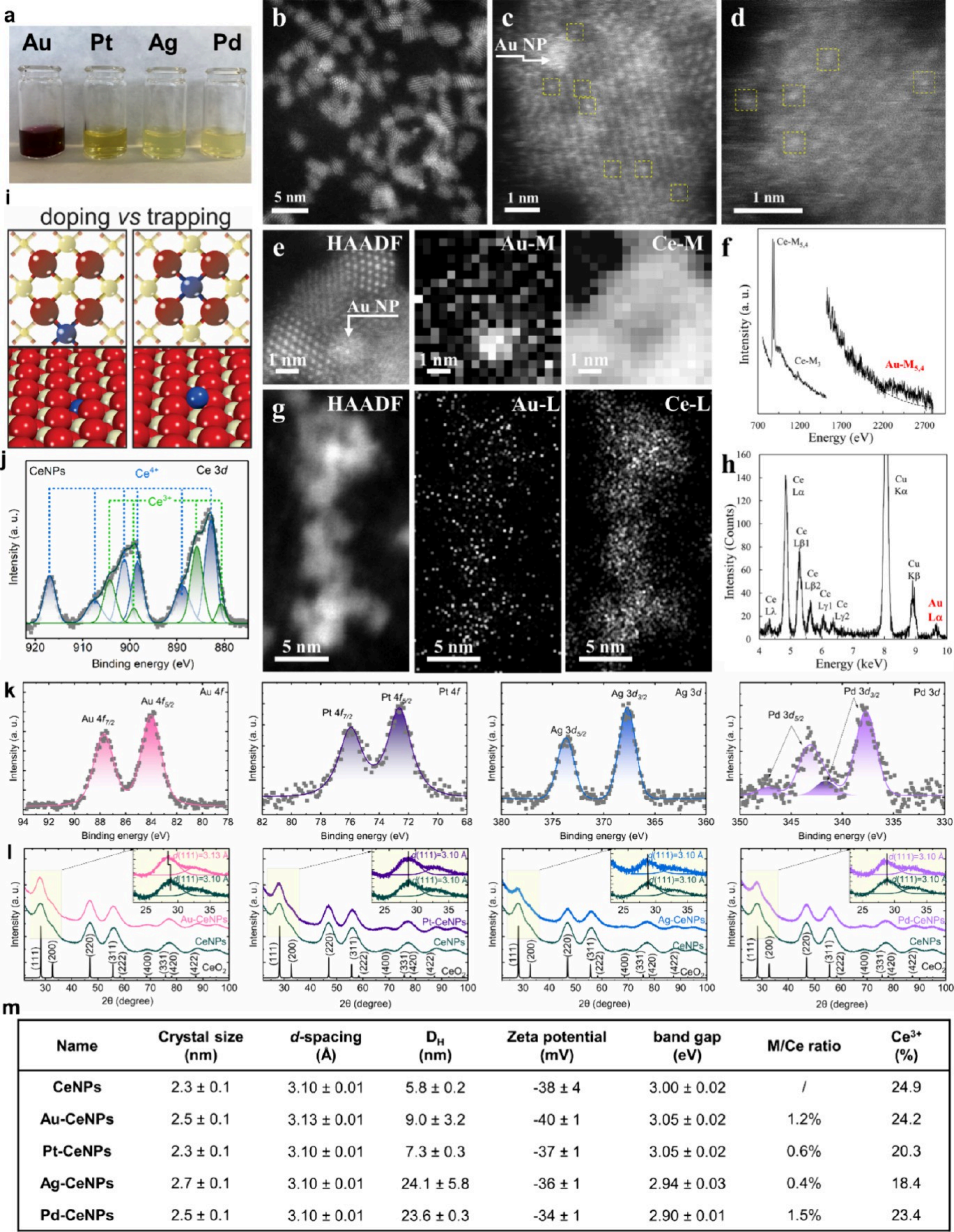
Physicochemical characterization of CeSAN-bots. (a) Visual appearance of Au-, Pt-, Ag-, and Pd-decorated CeNPs. (b) HAADF-STEM image of dispersed Au-CeNPs; HAADF-STEM of Pt-, Ag-, and Pd-decorated CeNPs are displayed in Figure S2. (c, d) High-resolution HAADF-STEM images of Au-CeNPs showing the presence of Au single atoms (marked by yellow squares); among them, an Au NP is identified (marked by arrows in (c) and (e)). (e) STEM-EELS elemental mapping is represented by a referential HAADF-STEM image and corresponding core-loss signal maps of Au-M and Ce-M edges. (f) EELS core-loss spectra collected from the area of the maps in (e) reveal signals of Ce-M and Au-M edges. The jump ratio of the Au-M signal is highlighted by a dashed curve. (g) STEM-EDXS elemental mapping is represented by a referential HAADF-STEM image and corresponding maps from net intensities of Ce-L and Au-L lines. (h) EDX spectra collected from the area of maps in (g) reveal the signals of Ce-L and Au-L lines (Cu K lines present in the spectra come from the TEM grid). (i) Proposed scheme of doped vs trapped metal components on CeO_2_(111). Color coding of atoms: red, O; beige, Ce; blue, metal. (j) Ce 3d core-level spectra of CeNPs. Ce 3d spectra of other NPs are shown in Figure S4. (k) Core-level spectra of Au 4f, Pt 4f, Ag 3d, and Pd 3d were obtained from XPS. Au 4f_7/2_ exhibits a binding energy of around 84.0 eV assigned to Au^δ+^. Pt 4f_7/2_ shows a binding energy of 72.6 eV indicating either a mixture of Pt^0^/Pt^2+^ or Pt­(OH)_2_. The Ag 3d_5/2_ peak has a binding energy of 367.7 eV that can be assigned to Ag^2+^. The Pd 3d doublet with a binding energy of 377.7 eV for Pd 3d_5/2_ may be ascribed to the Pd^2+^ species, while another smaller Pd 3d doublet with Pd 3d_5/2_ at 341.6 eV can be attributed to Pd^4+^ ions. (l) XRD patterns of Au-, Pt-, Ag-, and Pd-decorated CeNPs compared to CeNPs and CeO_2_ fluorite structure; the *d-*spacing of (111) was analyzed in the enlarged graph. (m) Table of physicochemical properties of Au-, Pt-, Ag-, and Pd-decorated CeNPs and CeNPs, including the crystal size and *d*-spacing of the (111) plane measured by XRD, the hydrodynamic diameter (*D*_H_ based on the number) and zeta potential measured by DLS, the band gap measured by UV–visible absorption spectroscopy, and the metal/Ce ratio and Ce^3+^/Ce percentage measured by XPS.

The high-resolution HAADF-STEM imaging was further utilized to characterize the Au doping in the case of the Au-CeNPs atomic structure. It revealed sparsely dispersed Au atomic clusters with a size of ∼1 nm among the CeNPs and, importantly, highly dispersed Au single atoms on the Au-CeNPs (Figure 2c,d and Figure S5). The observation of Au single atoms was possible due to the applied HAADF-STEM conditions creating the image predominantly from elastically scattered electrons by the Coulomb interactions (Rutherford scattering). This phenomenon allows for distinguishing atoms with different atomic numbers *Z* with the so-called *Z*-contrast. Since the contrast in the HAADF image is proportional to ≈*Z* ^2^, heavier _79_Au atoms appeared brighter in contrast with _58_Ce atoms in the HAADF-STEM images (light oxygen atoms were invisible for the used technique).^20^ In particular, the HAADF-STEM imaging revealed the atomic lattice structure of CeO_2_ nanocrystals containing visible brighter atomic positions, which can be addressed to the presence of single Au atoms. The CeNPs were lying randomly oriented on the carbon membrane, and the Au single-atom positions were often well-visible when the particles were oriented slightly out of a zonal axis. Many single Au atoms were observed spread and randomly positioned over the CeNPs, which agrees with the knowledge that Au does not form orderly atom alignment with the CeO_2_ crystalline structure.^21^ It should be noted that the STEM technique used did not allow us to determine whether the single Au atoms were localized on or inside the CeNPs.

To confirm the presence of the dispersed single Au atoms observed via HAADF-STEM imaging, two complementary spectroscopy measurements coupled with STEM were performed. Electron energy loss spectroscopy (EELS) and STEM-EELS elemental mapping were performed to acquire a spectral data-cube together with the high-resolution HAADF-STEM image of the Au-CeNPs atomic structure (Figure 2e). The resulting signal maps processed as Ce-M and Au-M edge intensities with subtracted background revealed the Au-M signal correlated with the shape of the imaged Au-CeNPs structure. Additionally, the Au atomic cluster was determined with the Au-M map. The Au-M edge EEL spectra integrated from the entire area of the collected data-cube (Figure 2f) further validate the presence of the Au doping of the CeNPs structure. The second complementary measurement used was energy-dispersive X-ray spectroscopy (EDXS) and STEM-EDXS elemental mapping. The HAADF-STEM image and corresponding signal maps processed as Ce-L and Au-L line net intensities with subtracted background revealed a dispersed Au-L signal correlated with the shape of the observed Au-CeNPs structure (Figure 2g). The detected dispersed Au doping via STEM-EDXS elemental mapping is supported with the EDX spectra of the Au-L line integrated from the volume of the observed Au-CeNPs structure (Figure 2h). This is an unambiguous proof of the presence of the dispersed Au doping of the CeNPs observed before via the HAADF-STEM. In addition to this STEM-EDXS qualitative analysis, a quantitative analysis of the EDX spectra was possible and it is presented in Figure S6. It determined the CeO_2_ stoichiometry and the amount of the Au doping in the concentration of 1.5 at. %, which corresponds with the Au concentration of 1.2 at. % as determined by XPS.

Cerium oxide has intriguing characteristics as a widely utilized metal oxide support capable of accommodating foreign elements. The accommodation mechanism can be categorized as (1) cation doping^22^ and (2) atomic trapping^19^ (Figure 2i). Cation doping involves substituting Ce^4+^ with foreign cations (such as Zr^4+^ or Gd^3+^) into the crystal lattice of CeO_2_. The introduction of cations with different ionic radii into the cerium oxide causes lattice strain changes, which disrupt the regular arrangement of oxygen atoms and promote the formation of oxygen vacancies. Alternatively, when noble metals are dispersed onto cerium oxide surfaces, they exhibit a pronounced preference to be trapped at the step edges and oxygen vacancies, which is attributed to the favorable energy landscape of these sites^23^ known as the strong metal–support interaction (SMSI).^24^ The two different mechanisms of impurity insertion can lead to notable alterations in the physicochemical properties of CeO_2_, considerably impacting its catalytic efficiency. In this work, the bandgap energy of M-CeNPs (M = Au, Pt, Ag, and Pd) and the *d-*spacing along the (111) direction remain unchanged compared to pristine CeNPs (Figure 2l and Figure S3), indicating no lattice expansions/contractions. Impurity crystalline phases originating from the metals were not observed. The Ce^3+^ fraction of M-CeNPs (Figure 2j and Figure S5) decreases slightly, ranging from 18 to 24%, compared to 25% for CeNPs. In contrast to elemental doping,^22^ noble metal modifications suppress surface defect formation. Figure 2k reveals that the valence state of deposited metals exists in their oxidized form due to their atomic dispersive nature on the oxide surface. The Au 4f_7/2_ peak, centered at 84.0 eV (upshift of 0.3 eV compared to metallic Au), can be ascribed to the slight positive charge on gold resulting from its atomic interaction with the cerium oxide support. Figure 2m summarizes the physicochemical properties of synthesized CeSAN-bots.

Based on the results obtained, we rule out lattice distortion and surface defect formation during these noble metal decorations on cerium oxide for assembling CeSAN-bots. The interaction of noble metal atoms with CeNPs follows the proposed atom-trapping pathway. We assume that atomically dispersed metal species preferentially sit in the square pockets of four oxygen ions and geometrically reside on top of the Ce cation sublayer, as has been confirmed in the case of Pt on CeO_2_,^23^ although further theoretical investigation is needed to verify the nature of the metal oxide support charge transfer.^19^

### Catalytic Activity of CeSAN-bots for Glucose Oxidation

The utilization of nanostructured gold in glucose oxidation has been reported as glucose oxidase mimetics.^25^ Figure 3a confirms that all synthesized M-CeNPs exhibited GOx mimetic activity as quantified by a H_2_O_2_-independent glucose assay kit (Note S2). Glucose oxidation activity is attributed to the anchored noble metal species on the CeSAN-bots. Glucose is oxidized to produce H_2_O_2_ in the presence of dissolved O_2_. Cerium oxide has been reported to act as a catalase-mimicking enzyme scavenging excessive H_2_O_2_.^26^ As shown in Figure 3b, the pristine CeNPs and M-CeNPs all exhibit catalytic activity for H_2_O_2_ disproportionation as catalase mimics.

**Figure 3:**
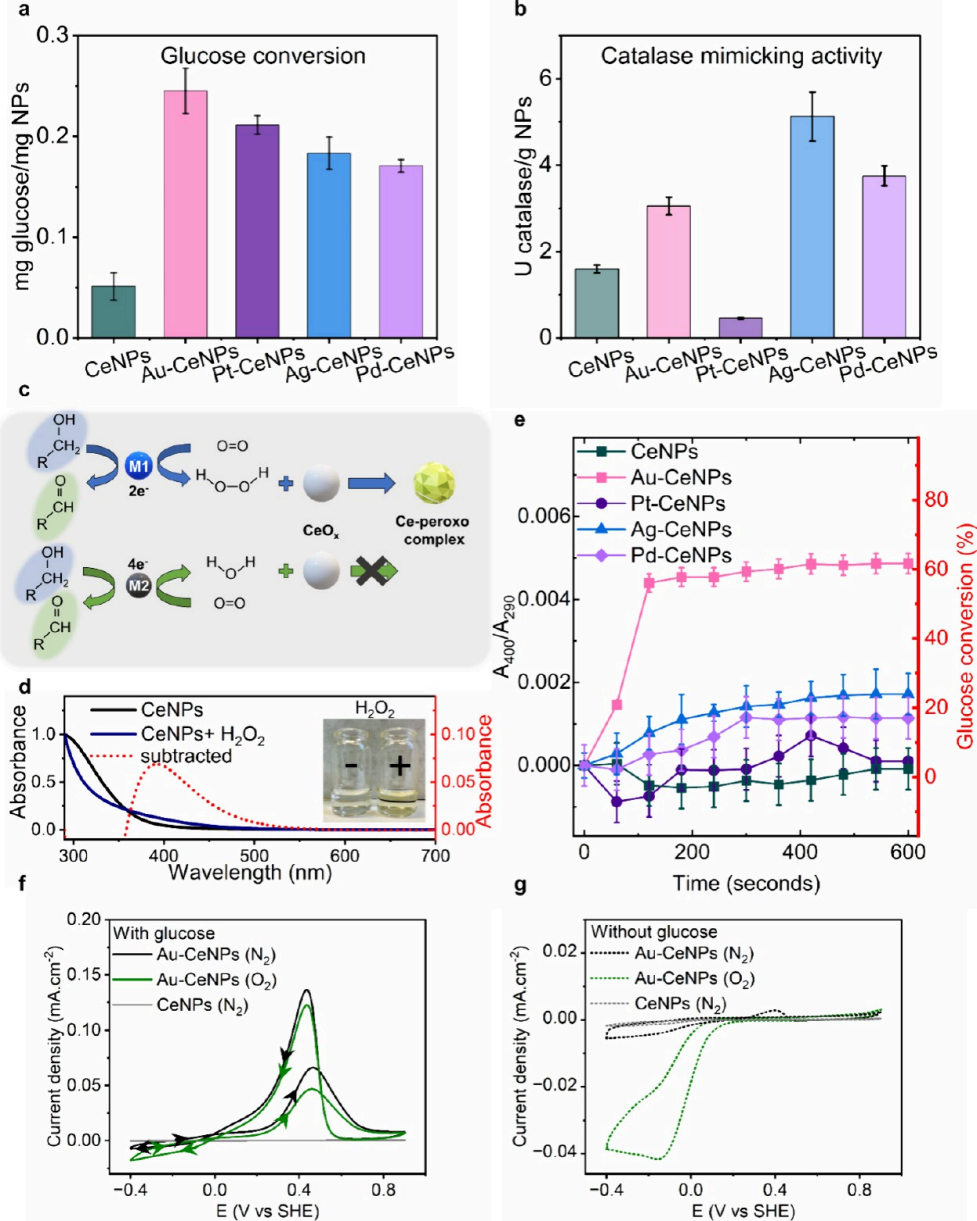
Catalytic activity of CeSAN-bots. (a) Catalytic oxidation of glucose by M-CeNPs as glucose oxidase mimetics quantitatively characterized utilizing hexokinase and adenosine triphosphate. (b) Catalytic reaction of H_2_O_2_ disproportionation by M-CeNPs as catalase (CAT) mimetics. (c) Proposed reaction schemes for coupled GOx and CAT mimics mediated by different metallic species, following either 2e or 4e transfer pathways. (d) UV–visible absorption spectra of pristine CeNPs colloidal solutions, CeNPs after H_2_O_2_ addition, and the presence of Ce-peroxo/hydroperoxo complexes as the reaction intermediate after spectra subtraction. The inset shows the color changes due to H_2_O_2_ addition. (e) GOx and CAT coupled reaction kinetics of different M-CeNPs. The absorption intensity of *A*_400_/*A*_290_ is used to represent the extent of glucose oxidation followed by H_2_O_2_ disproportionation of CeNPs. (f) Cyclic voltammograms (CV) of Au-CeNPs in N_2_- and ambient air-saturated (∼21% O_2_) 0.1 M KOH solutions with glucose at 20 g L^–1^. The CV curve of CeNPs in the N_2_-saturated KOH electrolyte is shown as a reference. Arrows indicate the sweeping direction. (g) Cyclic voltammogram of Au-CeNPs in N_2_- and ambient air-saturated (∼21% O_2_) 0.1 M NaOH solutions without glucose.

Noble metal-catalyzed glucose oxidation with O_2_ as an electron acceptor has been reported to proceed in two different pathways (Figure 3c). Depending on the number of electrons transferred during oxidation, O_2_ can be reduced to H_2_O_2_ (two-electron transfer pathway) mimicking natural GOx or directly reduced to H_2_O following the four-electron transfer pathway.^27^ By studying in detail the catalytic mechanism of glucose oxidation, Chen et al.^27^ have classified Au NPs into the two-electron transfer pathway while other noble metals as the four-electron transfer since they possess catalytic activity as peroxidase and oxidase mimics. Figure 3d shows the color changes of CeNPs from transparent to yellow upon addition of H_2_O_2_ addition. Colloidal CeNPs have a characteristic peak of around 290 nm. In the presence of H_2_O_2_, this peak disappears while absorption at 400 nm increases. By subtracting the original CeNPs spectrum from the one after H_2_O_2_, a differential spectrum with a peak at 400 nm was obtained (Figure 3d), which can be attributed to the stable reaction intermediate species called cerium-peroxo/hydroperoxo complexes rather than the long-attributed blueshift.^28^ We further applied the coupled reaction as indicated by the absorbance differentials *A*_400_/*A*_290_ to characterize glucose oxidation reaction kinetics (Figure 3e). Although the other three noble metal-coupled CeNPs (Pt-CeNPs, Ag-CeNPs, and Pd-CeNPs) catalyze glucose oxidation, no H_2_O_2_ is formed to react with CeNPs, as no increasing absorbance peak of cerium-peroxo/hydroperoxo complex formation is observed. This implies that the four-electron transfer catalytic pathway proceeds during the catalyzed glucose oxidation mediated by Pt-, Ag-, and Pd-CeNPs. Au-CeNPs achieve ∼60% glucose conversion within the first 3 min when coupled with CeNPs, further indicating their balanced coupling reaction between glucose oxidase and catalase mimetic activities where Au follows the two-electron pathway for glucose oxidation.

Conversely, natural GOx can transfer electrons not only from glucose to O_2_ but also to electrodes in electrochemical settings, facilitating its widespread application in glucose-powered enzyme fuel cells.^29^ Figure 3f shows the cyclic voltammetry (CV) curve for Au-CeNPs in the presence of glucose and a N_2_-saturated electrolyte; an obvious oxidation peak appeared around 0.45 V (versus SHE) as *E*_f_-glucose (f stands for forward), where glucose oxidation occurs. In the atmospheric air-saturated electrolyte (∼21% O_2_), the oxidation current decreased since the electrons collected by Au-CeNPs from glucose are competitively transferred to dissolved oxygen.^18^ In the absence of glucose under atmospheric conditions (Figure 3g), the peak between −0.4 and −0.1 V proves that oxygen can acquire electrons from the Au-CeNPs surface and conduct an oxygen reduction reaction (ORR), while in the less oxygen-saturated conditions, the ORR is significantly minimized. Another intriguing effect observed is the oxidation peak at the backward cathodic scan (*E*_b_, where b stands for backward). During the backward cathodic scan in the presence of glucose, a strong oxidation peak appears in the double-layer region (Figure 3f) only in Au-CeNPs but not in CeNPs.^30^ The effect of the oxidation peak on the cathodic scan was further investigated and is explained in Note S3 and Figures S7 and S8. Recent evidence suggests that *E*_b_ is closely related to the reduction of the metal oxide/oxyhydroxide layer, together with the desorption of the chemisorbed species.^31^ The cathodic scan exposes a fresh metal surface, leading to a second wave of glucose oxidation on the metal surface. The reactivated catalyst processes higher activity than that during the forward anodic scan, which leads to the hypothesis that the reduced species of Au are the true active site for glucose oxidation.

### “Sweet Swimmers”: Glucose-Powered CeSAN-bots

Numerous designs have reported effective propulsion based on GOx-mediated glucose oxidation^6,9,32,33^ due to the generated chemical gradient leading to self-diffusiophoresis. However, the question of whether it can be classified as ionic or neutral self-diffusiophoresis remains open for discussion.^34^ To characterize the motility of CeSAN-bots, we use dynamic light scattering (DLS)^35^ to quantify their diffusion coefficient since CeSAN-bots are below the detection limit of visual tracking techniques. As shown in Figure 4a, particle shape anisotropy allows the distinction of rotational diffusion due to intensity fluctuations arising from particle tumbling, although such an intensity fluctuation cannot be ruled out for particles that are spherical due to the optical anisotropy.^36^ The rotational diffusion relaxation time of Au-CeNPs in phosphate-buffered saline (PBS) solutions is 44 ± 6 μs, and the translational diffusion constant is 11.2 μm^2^ s^–1^. These values are in the range for the theoretically calculated rotational relaxation time for a 40 nm sphere based on the Einstein–Stokes equations (Note S4), corresponding to the measured intensity-based hydrodynamic diameter in the size of 40 nm, where its number-based hydrodynamic diameter is around 9 nm (further discussed in Note S5 and Figure S9).

**Figure 4:**
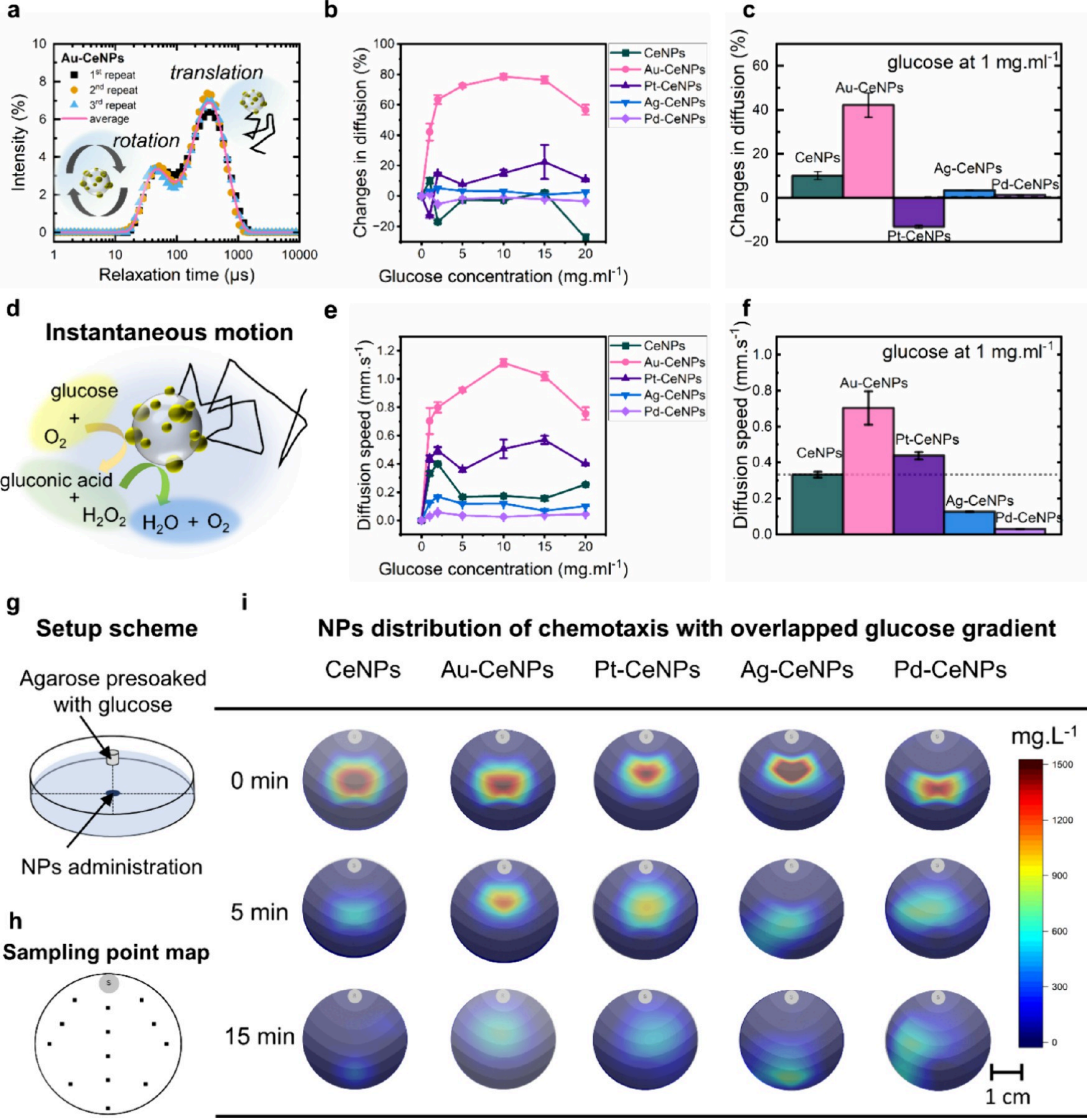
Glucose-mediated motion of CeSAN-bots. (a) Translational and rotational relaxation plot of Au-CeNPs in a PBS solution measured by DLS. (b) Percentage changes of diffusion constants as a function of glucose concentration for CeSAN-bots. (c) Percentage change of diffusion constants for CeSAN-bots at a glucose concentration of 1 mg mL^–1^. (d) Schematic representation of the self-diffusiophoresis of CeSAN-bots based on Au-CeNPs via the catalytic oxidation of glucose coupled with a catalase cascade reaction. (e) Average speed of CeSAN-bots as a function of glucose concentration. (f) Comparison of the average speed of CeSAN-bots at a glucose concentration of 1 mg mL^–1^. (g) Schematic setup of long-range chemotaxis mediated by glucose. A Petri dish with a cylindrical agarose gel presoaked with glucose was placed at the edge of the Petri dish and filled with PBS. Twenty g L^–1^ nanoparticles were administered at the center-bottom point of the Petri dish. (h) The concentration of NPs is sampled at different locations as indicated on the sampling map, at time intervals of 0, 5, and 15 min. The circle labeled “S” represents the position of the glucose source. (i) NP distribution heat maps were sampled at different time points. The 2D distribution heat maps of NPs overlap with the gray-shadowed isocratic lines showing a glucose gradient simulated by fluid dynamics.

The translational diffusion constants of Au-CeNPs show a significant increase with increasing glucose concentration (Figure 4b), which appears to be linear and saturates at 2 mg mL^–1^ glucose. At a physiological blood glucose level of 1 mg mL^–1^, Au-CeNPs show a four times greater enhanced diffusion coefficient compared to CeNPs (Figure 4c). For Pt-, Ag-, and Pd-based CeNPs, no significant increase in the diffusion coefficients is observed. The average speed of Au-CeNPs (calculation details in Note S4 ^35^) increases linearly with glucose up to 2 mg mL^–1^ (Figure 4e). At glucose concentration of 1 mg mL^–1^, Au-CeNPs reach a speed that is three times higher equivalent to ∼2.3 × 10^6^ body lengths/s compared to other M-CeNPs. It should be noted that due to the ultrasmall sizes of these nanoparticles, the rotational diffusion (Brownian motion) dominates over the movement, and these NPs quickly reorient, resulting in enhanced instantaneous diffusion, or active Brownian motion (Figure 4d), rather than directional movement. The calculated speed should be viewed with caution as an absolute value averaging over the Brownian motion, rather than the displacement of the particle position over time.

Self-diffusiophoresis can be observed from a macroscopic point of view as chemotaxis. We use a setup (Figure 4g,h) as previously reported to test the chemotaxis behaviors of nonbiological colloidal particles.^37,37^ The prebalancing time of glucose diffusion has been considered to minimize NP convection drifts caused by directional glucose diffusion (Note S6 and Figure S11). At 5 min, Au-CeNPs did not diffuse away; instead, the entire swarms of Au-CeNPs moved toward the source for a length of approximately 0.7 cm (Figure 4i), equivalent to a chemotactic velocity of 25 μm s^–1^. Pt-CeNPs show less obvious chemotaxis, while Ag-CeNPs and Pd-CeNPs behave like CeNPs where the nondirectional diffusion dominates. After 15 min, all particles diffused away. These data strongly support the observation that CeSAN-bots, particularly Au-CeNPs, exhibit long-range chemotactic behavior by following shallow gradients and converging toward the glucose source. This behavior occurred on a timescale of minutes and on a length scale 10^6^–10^7^ times greater than the size of the swimmers. Despite their initial directional movement toward the high-concentration region of glucose, CeSAN-bots do not exhibit long-term accumulation near the fuel source. Consequently, they underwent diffusion away from the high-concentration glucose region. Since CeSAN-bots lack strict Janus asymmetry that generates phoretic torque to correct misalignment of the catalytic domain with the chemical gradient,^38^ their chemotactic behavior involves a dependence on rotational diffusion times influenced by fuel concentration, resulting in accelerated chemokinesis and rapid dispersion^39,40^ (Note S7). These findings provide compelling evidence that CeSAN-bots possess glucose-mediated chemotaxis capabilities, significantly enhancing their efficiency in navigating within the physiological environment.

### Mechanistic Insights and Comparative Analysis of the Self-Propelled CeSAN-bots

Our results confirmed that among various precious metal-anchored CeNPs, only Au-CeNPs demonstrate self-diffusiophoresis and active Brownian motion, exhibiting glucose-responsive chemotaxis. The self-propulsion mechanism is attributed to the intrinsic interaction between gold single atoms and cerium oxide nanoparticles, which enhances their dynamic behavior. This section provides a detailed exploration of the underlying mechanism driving this self-propulsion and cross-reference comparison of CeSAN-bots with reported glucose-powered nanobots.

First, it is hypothesized that the enhanced motion comes from the unique interaction between gold atoms and cerium oxide nanoparticles. The enhanced motion of CeSAN-bots originates from the unique interaction between gold single atoms and CeNPs. This interaction occurs at the atomic level, where Au atoms are anchored on the surface of CeNPs, creating localized atomic heterogeneity. This process is fundamental to the self-propulsion mechanism. The catalytic mechanism involves a tandem reaction: first, glucose is oxidized by gold atoms to produce hydrogen peroxide (H_2_O_2_), which is then disproportionated by CeNPs. This two-step sequence is crucial for generating the product gradient necessary for self-diffusiophoresis. As shown in Figure 4d, catalytic activity alone, such as glucose oxidation by Pt, Ag, or Pd, is insufficient to generate propulsion unless coupled with H_2_O_2_ disproportionation. The cascading reactions create a product gradient that drives the motion of the CeSAN-bots.

The self-propulsion of CeSAN-bots relies on neutral self-diffusiophoresis, a mechanism in which motion is driven by the product gradients generated by the catalytic reactions.^35,41–43^ Unlike ionic self-diffusiophoresis, in which ion production is involved, neutral self-diffusiophoresis does not rely on the presence of ions. To confirm this, we measured the motion of Au-CeNPs in water and PBS solutions with varying ionic strengths (Figure S10). Despite significant changes in ionic strength, the diffusion speed remained constant, supporting that CeSAN-bots move through neutral self-diffusiophoresis, where the catalyst-induced product gradient, rather than ionic gradients, drives the propulsion.^44–46^

A critical factor in the self-propulsion of CeSAN-bots is the anisotropic atomic heterogeneity of gold atoms on the cerium oxide surface. This surface-level heterogeneity enhances the catalytic efficiency and facilitates slip velocity, which is essential for self-propulsion. Unlike bulk catalytic modifications, this atomic-level asymmetry is crucial for generating sufficient surface-related slip velocity, allowing CeSAN-bots to exhibit enhanced diffusion in response to glucose. To verify this hypothesis, we further synthesized Fe-CeNPs following a similar procedure, with the physiochemical characterization of Fe-CeNPs shown in Figure 5. The HR-STEM image of the brownish Fe-CeNPs colloidal solution (Figure 5a) shows that the synthesized Fe-CeNPs exhibited a nonregular shape of crystallites (Figure 5c), which is further confirmed by the lack of CeO_2_ fluorite structure in XRD (Figure 5e). The reduced CeNPs crystal sizes in the presence of Fe can be attributed to the replacement of Fe in the CeO_*x*_ crystalline structure resulting in crystallite distortion (Figure 5d). It has been reported that doping CeO_2_ with Fe^3+^ can hinder grain growth,^47^ which supports the observance of decreasing crystallite sizes. The final synthesized Fe-CeNPs show that Fe exhibited a 98% atomic ratio compared to Ce, indicating that 50% of Ce is replaced by Fe in the unit cell. Fe existed in its Fe^3+^ form (Figure 5f), forming strong oxygen bonds with the lattice oxygen in the cerium oxide crystalline structure (Figure 5d). The Ce^3+^/Ce ratio also increased significantly because of Fe doping (Figure 5g). The structure changes were further reflected by the decreased crystallite sizes (two times decreased compared with CeNPs) and *d*-spacing shrinkage due to the crystalline structural distortion. The indirect bandgap energy of Fe-CeNPs is smaller than that of CeNPs, similar to the previous report after Fe modification.^47^ Fe-CeNPs exhibited an extra bandgap energy below the observed typical bandgap energy for CeNPs. As described by Makula et al.,^48^ such an intrabandgap state usually results from defective, doped, bulk, or surface-modified materials. Based on all the physicochemical characterizations, Fe-CeNPs exhibited a distinct opposite trend of modification compared to the noble metal-decorated M-CeNPs, further indicating that the final product should be expressed as the FeCeO_*x*_ composite, where Fe decorated CeNPs structures by elemental cation doping rather than atomic trapping as in the case of Au-CeNPs (Figure 5b). Based on the results presented in Figure 5h, Fe-CeNPs exhibited enhanced catalytic activity toward glucose oxidation coupled with H_2_O_2_ disproportionation, and it is observable that this effect is much stronger than that of Au-CeNPs. We further analyzed the motion behavior of the Fe-CeNPs. Figure 5i,j indicates that Fe-CeNPs did not exhibit enhanced diffusion in the presence of glucose. This is further confirmed by the lack of chemotaxis of Fe-CeNPs toward the external glucose gradient (Figure 5k). Although Fe-CeNPs show tandem glucose-H_2_O_2_ catalytic activities comparable to those of Au-CeNPs (meeting criterion 1), there is no observable enhanced diffusion or observable chemotaxis. The static behavior of Fe-CeNPs is attributed to the fact that elemental doping results in a lack of sufficient surface-related slip velocity.

**Figure 5:**
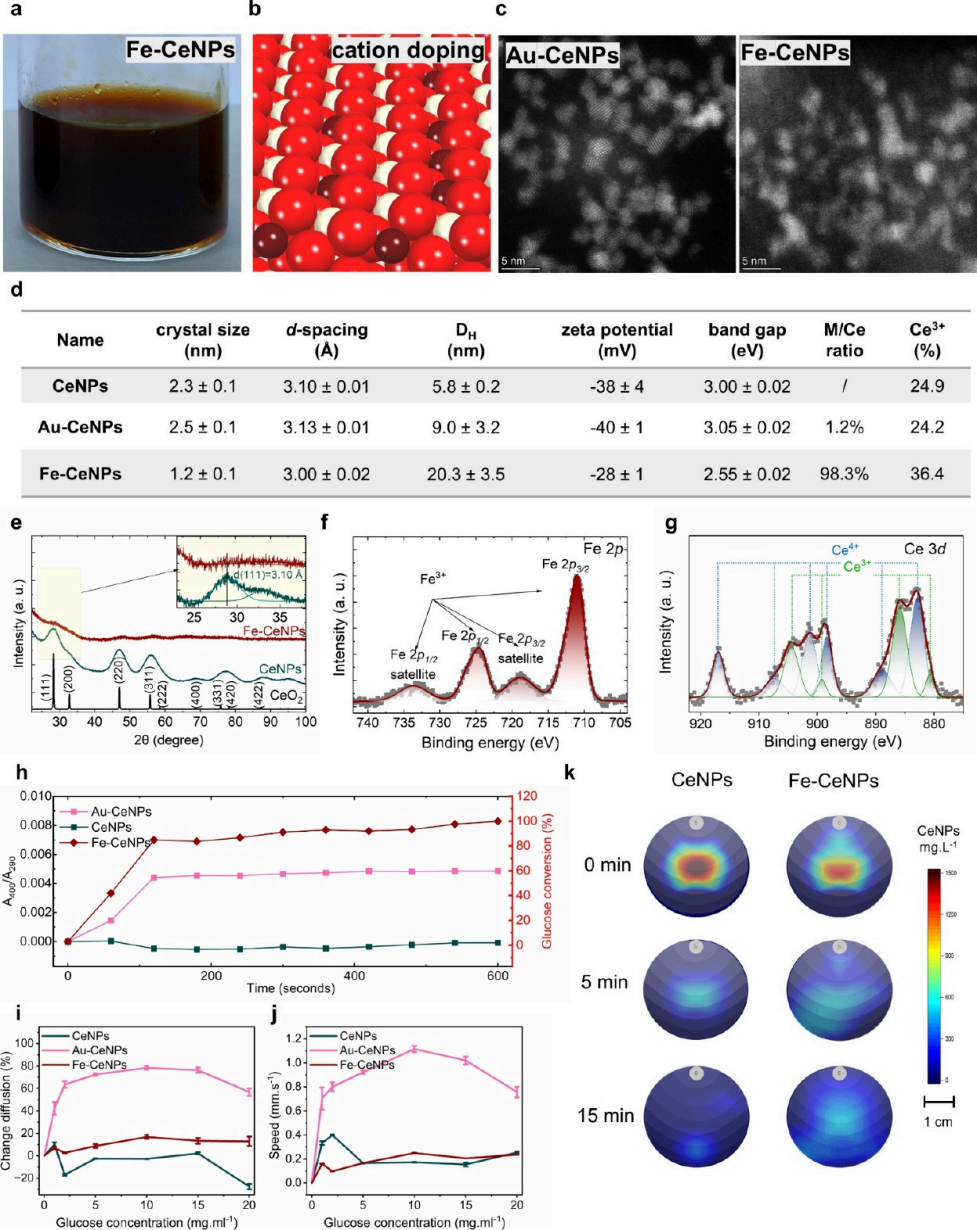
Fe-CeNPs physicochemical characterization and motion analysis. (a) Stable Fe-CeNPs colloidal solution. (b) Elemental cation-doped structure on cerium oxide. Color coding of atoms: red, O; beige, Ce; brown, Fe. (c) HAADF-STEM images of Au-CeNPs and Fe-CNPs. (d) Table of physicochemical properties of synthesized Fe-CeNPs compared to CeNPs and Au-CeNPs. (e) XRD patterns of Fe-CeNPs compared to CeNPs and CeO_2_ fluorite structure. The *d-*spacing of (111) was analyzed in the enlarged graph. (f) Fe 2p core-level spectra. (g) Ce 3d core-level spectra. (h) GOx and CAT coupled reaction kinetics of CeNPs, Au-CeNPs, and Fe-CeNPs. The absorption intensity of *A*_400_/*A*_290_ is used to represent the extent of glucose oxidation followed by H_2_O_2_ disproportionation of CeNPs. (i) Percentage change of diffusion constants as a function of the glucose concentration for CeNPs, Au-CeNPs, and Fe-CeNPs. (j) Average speed of CeNPs, Au-CeNPs, and Fe-CeNPs as a function of the glucose concentration. (k) NP distribution heat maps due to chemotaxis sampled at different time points. The 2D distribution heat map of NPs overlaps with the gray-shadowed isocratic lines showing the glucose gradient simulated by fluid dynamics. CeNPs and Fe-CeNPs were presented for comparison.

This observation supports the glucose-mediated propulsion mechanism of CeSAN-bots, which depends on two critical criteria: (1) glucose catalytic nanozymes enabling tandem reactions with cerium oxide for H_2_O_2_ disproportionation and (2) surface anisotropic atom heterogeneity, rather than lattice incorporation. Our work also provides further insight into the recent debate of whether the Janus structure is essential for enzyme (like) powered micro/nanomotors.^49,50^ We suggest that a distinct catalytic asymmetry may not be crucial for metal–catalyst-based propulsion but rather that inherent atomic-level heterogeneity plays a pivotal role in generating self-propulsion.

Table S1 provides a comprehensive comparison of reported glucose-powered nanorobots utilizing similar mechanisms of action, including parameters such as size, propulsion mechanisms, and performance metrics. These are benchmarked against the current enzymeless CeSAN-bot system, highlighting its advantages in efficiency and design simplicity. For glucose oxidase (GOx)-powered micro/nanomotors using glucose as fuel, existing designs in the literature focus mostly on immobilizing enzymes (GOx) onto various surfaces.^51^ It starts from the early models such as conductive carbon fiber utilizing electron current propulsion^52^ and the self-assembled monolayer in a microfluid pump with density-driven convective flow.^53^ More recent strategies for the design of glucose-based micro/nanomotors involve the incorporation of GOx,^54^ coupled with other enzyme cascade reactions (such as catalase to convert the byproduct of H_2_O_2_ to nontoxic H_2_O),^9^ into vectors such as Janus silica nanoparticles,^6,32,33^ polymersomes,^37^ liposomes,^55^ and even biological cells such as stomatocytes.^8^ The reported coupling pathway involves the trapping of GOx in confined spaces,^8,37,52,53,56^ employing electrostatic adsorption,^33,53^ or utilizing covalent cross-linking conjugation (such as glutaraldehyde,^6,32^ EDC/NHS,^57^ streptavidin–biotin,^58^ etc.). However, these methods often result in a compromise in GOx activity^6^ due to a disrupted enzyme structure^33^ or limited exposure of the active sites^8^ necessary for efficient glucose conversion. When comparing the velocity of GOx enzyme-functionalized glucose micromotors, typically ranging from a few hundred nanometers to several micrometers in size with velocities between 10 and 100 μm s^–1^ as listed in Table S1, the current study reports a significant advancement: nanomotors smaller than 10 nm achieving apparent velocities in the millimeter-per-second range. However, it is important to note that a particle’s apparent velocity is directly influenced by its size. As the particle size decreases, its diffusion coefficient increases, resulting in a more pronounced Brownian motion. Therefore, direct velocity comparisons across motors with different sizes are inherently limited and can be misleading. A few more recent studies focused on utilizing Au nanoparticles for glucose conversion, a similar concept as applied in this study.^11,59^ However, the fabricated structures are either too complicated with potential toxicity (high metallic content) or very limited to specific biomedical applications (with generation of NO bubbles). Among all reported glucose-powered nanomotors, whether biohybrid (enzyme-based) or metal-based, the CeSAN-bots presented in this study are the smallest in size going sub-10 nm. They exhibit a significant enhancement in diffusion coefficient (>80%), high apparent velocity, and exceptional chemotactic velocity, positioning them as promising candidates for biomedical applications.

### CeSAN-bots Enhance Stem Cell Regeneration

In ophthalmology, MSC therapy shows promise in treating ocular surface injuries by suppressing inflammation and promoting the regenerative microenvironment. Despite the documented benefits in corneal wound healing, preclinical evaluations pose some uncertainties about the long-term fate of MSCs due to their susceptibility to oxidative stress.^60^ CeSAN-bots, with their catalase and superoxide dismutase mimicking antioxidant activity, could potentially support the survival of MSCs and promote their differentiation and migration. Our *in vitro* study testing the effects of cerium oxide-based CeSAN-bots shows increased metabolic activity of exposed MSCs (Figure 6a). This increase in metabolic activity, as measured by the WST-1 assay, directly correlates with enhanced MSC proliferation, confirming the positive impact of CeSAN-bots on cell growth. Given that the WST-1 assay provides a reliable and widely accepted method for assessing cell proliferation, these results strongly support the role of CeSAN-bots in promoting the MSC expansion. Confocal microscopic images (Figure 6b) show that CeSAN-bots@DiI (labeled with a fluorescent DiI stain) are internalized by the MSCs and located primarily in the cytoplasm, as reported previously.^17^ Fast internalization of Au-based CeSAN-bots to MSCs is demonstrated in Figure 6c. Within the first hour, Au-CeNPs exhibit at least a two-fold faster uptake than CeNPs (Figure 6d). The enhanced uptake is attributed to active diffusion facilitated by the glucose influx from extracellular to intracellular space. Next, we examined whether MSCs preserve their immunomodulatory effects^61^ after CeSAN-bots internalization (Figure 6e). The low adverse effect of CeSAN-bots on MSCs is verified by no induction of expression of p53 (rather downregulation), whose protein product is an important regulator of apoptosis. A mild upregulation of nerve growth factor (NGF) expression and a significant decrease in hepatocyte growth factor (HGF) production in MSCs treated with CeSAN-bots support that CeNPs can direct MSCs to a more regenerative phenotype and accelerate corneal healing (HGF can promote undesirable growth of the aberrant epithelium,^62^ while NGF supports proper cornea reepithelization^63^). The changes in programmed death-1 (PD-L1) expression are slightly upregulated, although, in most cases, it is statistically insignificant. During the early stage of corneal damage, leukocytes infiltrate the site of injury and macrophages are activated to a proinflammatory phenotype, causing the release of inflammatory cytokines. The production of interleukin-6 (IL-6) significantly decreased after the internalization of CeSAN-bots by MSCs. The production of IL-6 is related to the induction of proinflammatory T helper 17 (T_h_17) lymphocytes.^64^ As concluded in Figure 6f, specifically, CeSAN-bots can potentially inhibit cellular inflammation, creating an immunotolerant-favorable environment and consequently amplifying MSC regenerative potential. The hybrid system of CeSAN-bot-incorporated MSCs, in contrast to MSCs alone, demonstrates a more powerful cell regeneration and immunomodulatory therapeutic effect, further addressing the vulnerability of MSCs to harsh environmental exposure.

**Figure 6:**
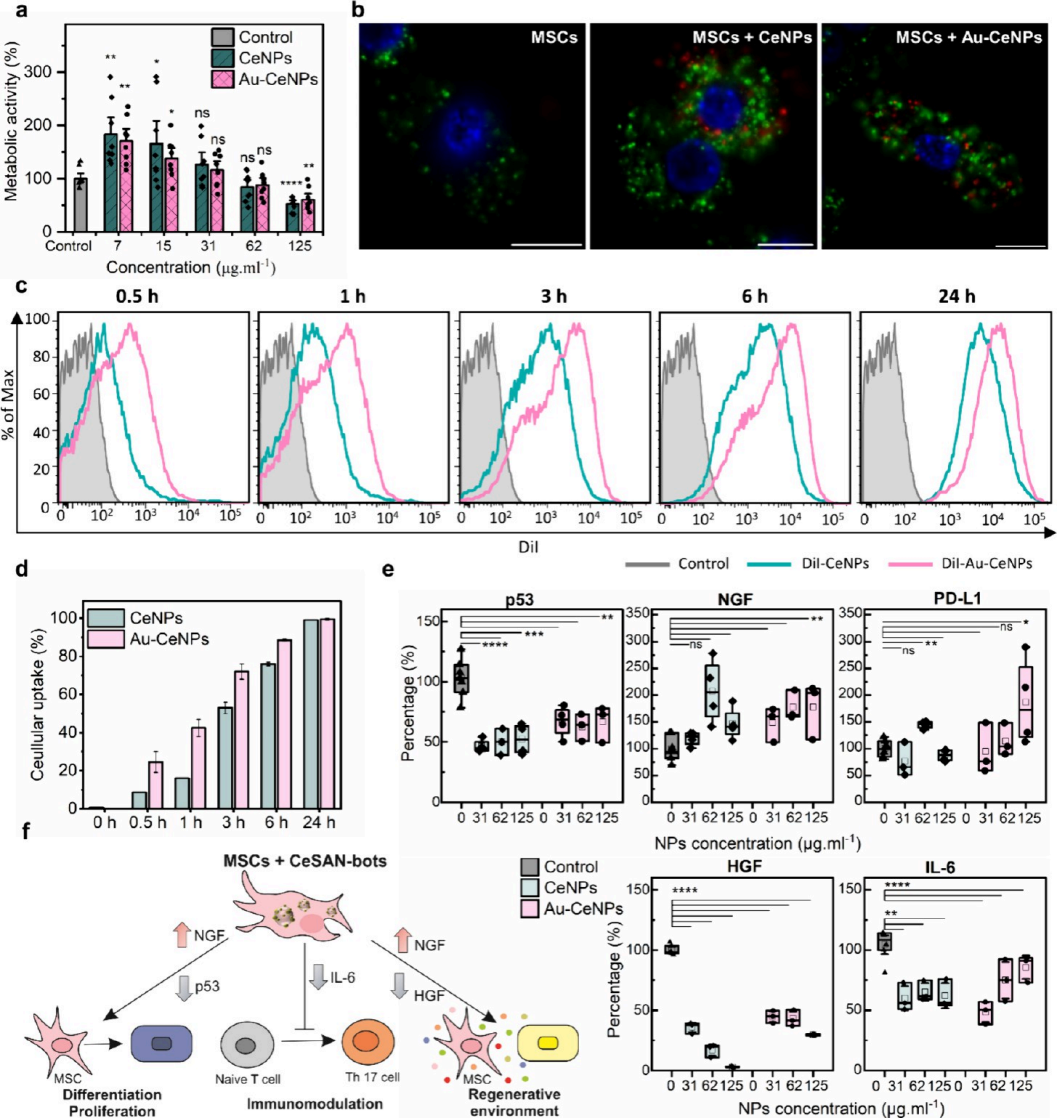
*In vitro* studies of the influence of CeSAN-bots on MSCs. Four independent experiments were conducted. Data were presented as means ± s.e.m. *P* values were analyzed by a two-sample *t* test, where *P < 0.05, **P < 0.01, ***P < 0.001, and ****P < 0.0001. (a) Metabolic activity of MSCs after incubation with CeSAN-bots (CeNPs and Au-CeNPs) assessed by the WST-1 assay. (b) Confocal microscopy image of the localization of CeSAN-bots@DiI (red) in PKH67-stained MSCs (green). Cell nuclei are stained with DAPI (blue). The scale bar represents 10 μm. One representative image for each type of sample is shown, magnification: 1000× (immersion objective). (c) Representative histograms showing the internalization of CeSAN-bots@DiI (63 μg mL^–1^) by MSCs analyzed by flow cytometry. One of three similar experiments is shown. (d) Flow cytometry quantification of the CeSAN-bots@DiI internalized by MSCs at different time points (between 0.5 and 24 h). (e) Effect of CeSAN-bots on MSC expression of genes for p53, NGF, and PD-L1 analyzed by real-time PCR and on the production of IL-6 and HGF analyzed by ELISA. The amount of HGF and IL-6 was acquired as optical density and, according to cytokine standards, was calculated as concentration in pg mL^–1^ and then converted to % compared with the control of the cytokine production level from untreated MSCs. (f) Schematic representation showing the potential mechanisms of regulation of immunomodulatory and regenerative MSC properties by CeSAN-bots.

In our study, the CeSAN-bots first utilized glucose from cell culture media as their energy source to enhance cellular uptake. Once internalized into the cells, it might continue to consume the endogenous glucose. To address concerns regarding potential impacts on cellular glucose metabolism, we conducted gene expression analysis focusing on key enzymes and factors involved in glucose metabolism such as pyruvate dehydrogenase (PDH) and hypoxia-inducible factor 1-alpha (HIF-1α). The PDH enzyme converts pyruvate into acetyl-CoA, fueling the tricarboxylic acid (TCA) cycle, while HIF-1α regulates metabolic adaptation by promoting glycolysis and inhibiting PDH under hypoxic conditions, reducing TCA cycle activity. Figure S12 showed no significant changes in their expression levels when MSCs were incubated with Au-CeNPs and CeNPs for up to 2 days. These results indicate that glucose utilization by CeSAN-bots does not disrupt critical metabolic pathways, including the TCA cycle. Moreover, the metabolic activity of MSCs remained stable during exposure to CeNPs and Au-CeNPs for up to 6 days (Figure S12c). In summary, CeSAN-bots utilize glucose from the culture media and potentially endogenous glucose after internalization without disrupting the TCA cycle or cellular metabolism, as confirmed by unchanged PDH and HIF-1α expression and stable MSC metabolic activity.

### Immunoregulatory Effects of CeSAN-bots under Inflammatory Environments

The above-mentioned experiments demonstrated enhanced cellular uptake and immunomodulatory effects of CeSAN-bots in a rested cellular environment. Following these results, we further evaluated how CeSAN-bots regulate cellular behavior *in vitro* at induced inflammatory environments. The ability of CeSAN-bots to scavenge excessive reactive oxygen species (ROS) was demonstrated with H_2_O_2_-induced oxidative stress on MSCs. Figure 7a shows that CeNPs can reduce the intracellular ROS level by 17% while Au-CeNPs achieved a 45% ROS reduction, demonstrating their potential as ROS scavenging antioxidants in inflammatory environments.

**Figure 7:**
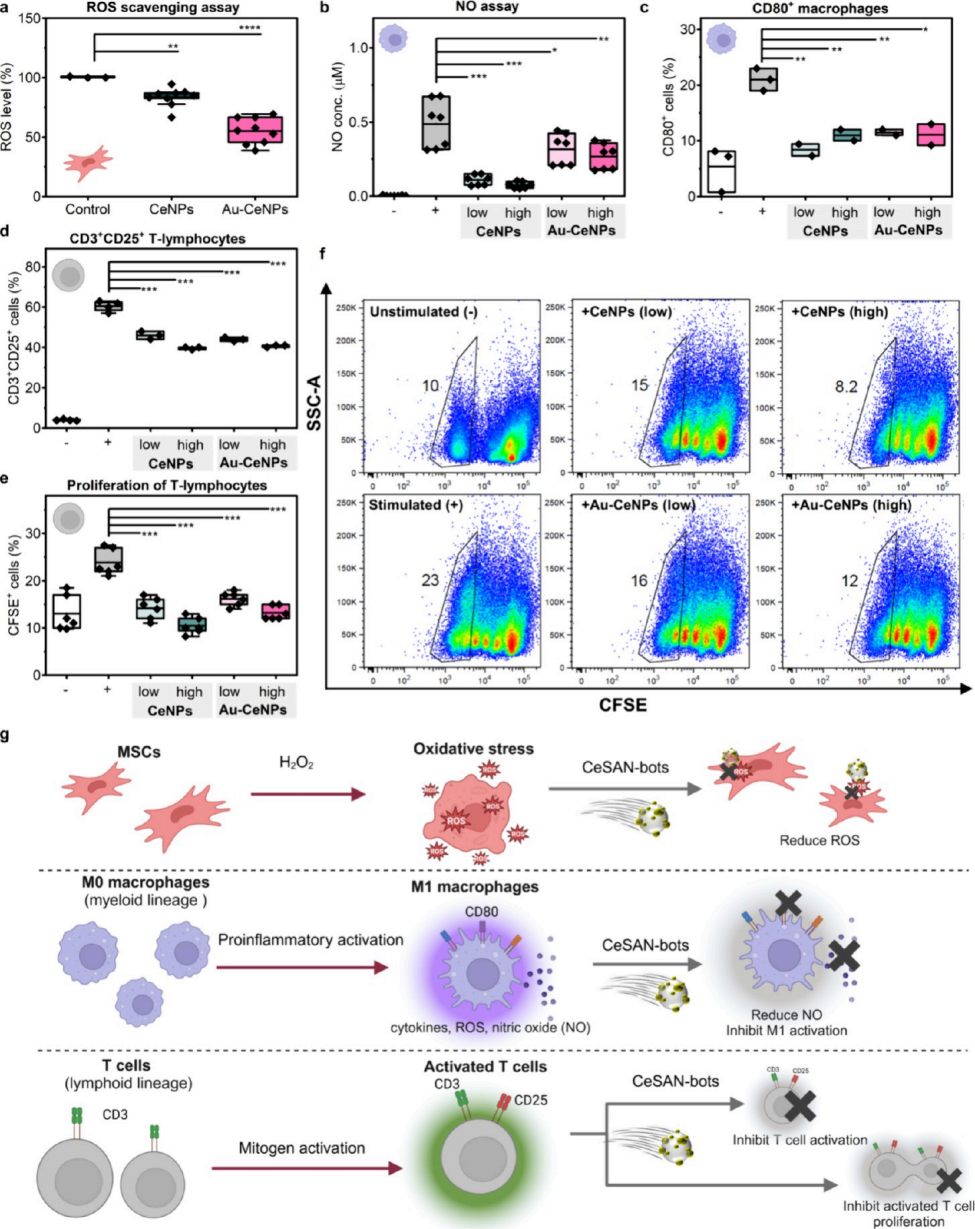
CeSAN-bots regulate the inflammatory environment at the cellular level. Box plots show data distribution (black diamonds) with the interquartile range (25–75%), the mean value represented by a horizontal line, and whiskers extending up to 1.5 times the quartile range. *P* values were analyzed by a two-sample *t* test, where *P < 0.05, **P < 0.01, ***P < 0.001, and ****P < 0.0001. (a) Measurement of intracellular ROS in MSCs (using DCDHF-DA) by flow cytometry assessed in three independent experiments. The amount of NPs used was 62 μg mL^–1^. (b) Effect of CeSAN-bots on the production of nitric oxide by activated macrophages assessed by the Griess reaction. Results from three independent experiments. The quantity of NPs used was 62 μg mL^–1^ for low amounts and 125 μg mL^–1^ for high amounts (the same conditions for the following graphs). (c) Effect of CeSAN-bots on the percentage of CD80^+^ activated macrophages assessed by flow cytometry. Results from two independent experiments. (d) Effect of CeSAN-bots on the percentage of activated CD3^+^CD25^+^ T-lymphocytes assessed by flow cytometry. Results from three independent experiments. (e) Effect of CeSAN-bots on the proliferation of CFSE-labeled mitogen-stimulated T-lymphocytes assessed by flow cytometry. The presented result is the average of three independent experiments. (f) Representative flow cytometry dot plots showing a decrease in percentage of proliferating CFSE-labeled T-lymphocytes by CeSAN-bots. Plots represent one of the three independent experiments. (g) Schematic representation illustrating the ROS scavenging ability and immunomodulatory effects of CeSAN-bots in inflammatory environments at the cellular level.

Macrophages make up a crucial population of leukocytes essential for proper immune function. Under inflammatory conditions, resting M0 macrophages can polarize into M1 or M2 phenotypes, with M1 driving proinflammatory responses and M2 promoting anti-inflammatory and tissue repair functions. Activated macrophages contribute to inflammation by producing nitric oxide (NO), ROS, and proinflammatory cytokines, which amplify immune responses and recruit additional immune cells. However, prolonged activation may lead to tissue damage and chronic inflammation. To evaluate the immunomodulatory effects of CeSAN-bots on activated macrophages, we induced macrophage activation using lipopolysaccharide and interferon-γ.^65^ Figure 7b shows that both high and low concentrations of NPs (62 and 125 μg mL^–1^) inhibit NO production in activated macrophages. Additionally, we examined the regulation of activated macrophage populations by tracking CD80, a key costimulatory molecule and marker of macrophage activation. CeSAN-bots significantly reduced the percentage of activated macrophages by 50%, effectively suppressing the externally induced proinflammatory environment of macrophages.

The activation of T-lymphocytes is another critical component of the immune response. A mitogen was applied as a stimulator to achieve T-lymphocyte activation, as indicated by the increased population of CD3^+^CD25^+^ cells, where the coexpression of CD3 and CD25 serves as a marker of activated T-lymphocytes.^66^ CD3 is a component of the T-cell receptor (TCR) complex, signifying the presence of T-lymphocytes, while CD25, the alpha chain of the IL-2 receptor, is upregulated upon T-cell activation, reflecting their proliferative and functional state. Figure 7d shows a significant reduction of activated T-cell population after incubation with CeNPs and Au-CeNPs (∼25%). The effect on the proliferation of mitogen-stimulated T-lymphocytes is another key indicator of the immunomodulatory properties of CeSAN-bots (Figure 7e). T-lymphocyte proliferation was assessed by flow cytometry, where CFSE-labeled lymphocytes diluted the dye with each division (Figure 7f). Cells with more divisions show lower fluorescence, appearing further left, indicating higher proliferative activity. All the NPs significantly reduced the proliferation of mitogen-stimulated T-lymphocytes, returning their levels to prestimulation state. Figure 7g summarizes the modulatory effect of CeSAN-bots on stem cells and immune cells under an activated inflammatory environment at the cellular level. CeSAN-bots exhibit ROS and NO scavenging activity to protect stem cells from oxidative stress, along with immunoregulatory effects that suppress the overactivation and proliferation of both myeloid and lymphoid immune cells. Koo et al. reported an approach to apply MSCs as CeNPs carriers targeting inflammatory rheumatoid arthritis.^67^ It corresponds well with our observation that CeNPs-based nanoparticles can alleviate inflammation and modulate the tissue environment into a more immunotolerant state by bridging the innate and adaptive immunity. Our approach of utilizing CeSAN-bots for stem cell therapy offers a potential solution for treating damaged corneas by enabling MSCs, incorporated with nanorobots, to be applied to the targeted area for an extended period, thereby combating the inflammatory environment.

### CeSAN-bot-Incorporated Stem Cell Therapy for Corneal Injury

We further employed a mild corneal alkali burn model (grade I) in mice to investigate the therapeutic potential of CeSAN-bot-integrated MSCs *in vivo*. Four groups of mice (six mice in each group) were injected intravitreally with PBS (untreated), MSCs, CeNP-incorporated MSCs (MSCs+CeNPs), or Au-CeNP-incorporated MSCs (MSCs+Au-CeNPs). A detectable number of CeNPs and Au-CeNP-incorporated MSCs remained in the cornea even after 7 days (Figure 8a,b; the flow cytometry gating strategy is presented in Figure S13). Analysis of gene expression (Figure 8b) reveals increased expression of the gene for proinflammatory cytokine IL-6 due to the damaged cornea, which was downregulated in CeNP-incorporated MSC-treated mice. Additionally, CeNP-incorporated MSC treatment results in a significant increase in keratin 12 (K12) expression, a marker of the corneal epithelium, reaching almost K12 expression in healthy corneas. Insulin-like growth factor 1 (IGF-1), a key mediator of corneal regeneration, is observed to be upregulated in both CeNP-incorporated MSCs and Au-CeNP-incorporated MSC-treated groups. Furthermore, the Bax/Bcl ratio, an indicator of the apoptosis rate,^68^ is reduced in both NP-incorporated MSC-treated mice, indicating downregulation of apoptosis by treatment. Flow cytometric analysis of immune cell infiltration of the damaged eye reveals a reduction in the percentage of F4/80^+^ macrophages in both NP-incorporated MSC-treated mice. This is further supported by the results of analysis of the extract from the damaged eye, demonstrating decreased NO production reflecting a reduced number of activated macrophages (especially for CeNPs+MSCs). The average number of MSCs migrating to the damaged site is indicated by the number of PKH67^+^ cells shown in Figure 8b. The number of Au-CeNP-incorporated MSCs migrating to the damaged cornea is almost doubled compared to MSCs alone or CeNP-incorporated MSCs. The PKH67 labeling method is crucial for accurately tracking MSC migration to the cornea, as it enables specific, real-time visualization and quantification of MSCs within the targeted tissue. This assay provides valuable insights into MSC behavior and migration in the complex corneal microenvironment, offering a more precise and reliable measure than other commonly used migration assays such as transforaminal migration or wound healing assays. Corneal opacity scores were assessed according to the Roper–Hall criteria^69^ on day 7 after the application of treatment (Figure 8b,c). On day 0, all groups exhibit a corneal opacity score of around two, demonstrating the successful establishment of the model. In the CeNP-incorporated MSC-treated group, the degree of corneal opacity was significantly reduced from mild turbidity to near transparency, with an opacity score approaching one. Over 7 days, the repair rate of CeNP-incorporated MSC-treated mice improved by more than 30%, confirming the synergistic effect of CeSAN-bots in enhancing the therapeutic efficacy of MSCs. Although the opacity score improvement is absent in the case of Au-CeNP-incorporated MSCs, Au-CeNPs not only demonstrated accelerated cellular uptake by MSCs but also exhibited enhanced infiltration of MSCs into damaged corneal tissues. Furthermore, they improved the immunomodulatory and regenerative properties of the exposed MSCs. While CeNP-incorporated MSCs proved effective in improving the opacity score of the damaged cornea, the precise mechanism remains undisclosed for now. Our future work will involve a comprehensive whole genome analysis to compare MSCs loaded with CeNPs and Au-CeNPs, sorted from the damaged cornea, aiming to elucidate the specific changes induced in MSCs that enhance their therapeutic efficacy in damaged cornea repair.

**Figure 8:**
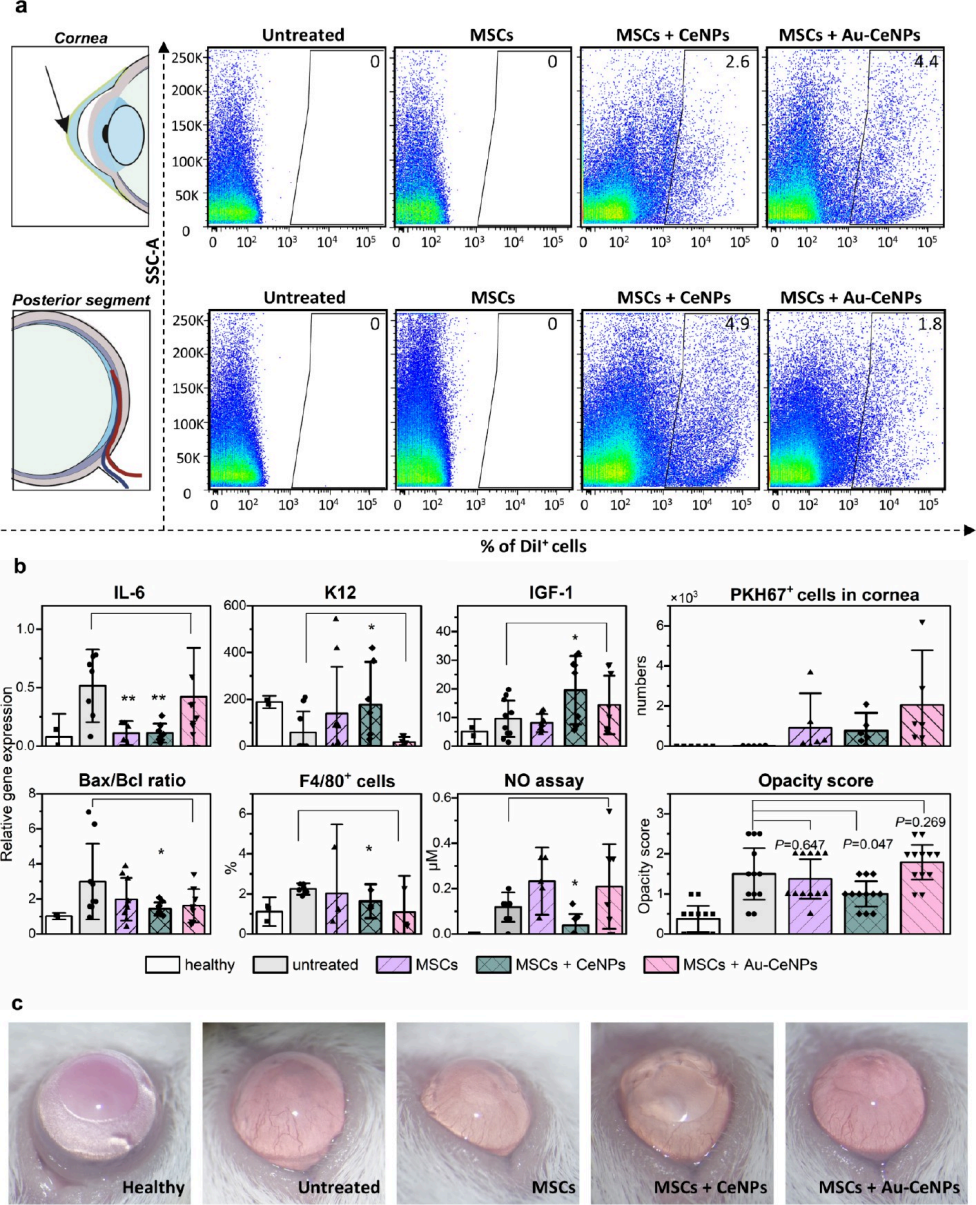
*In vivo* study of MSC therapy for treating corneal damage by CeSAN-bot-incorporated MSCs. (a) Dot plots from flow cytometry analysis representing the percentage of cells incorporated with CeSAN-bots@DiI detected in the cornea (upper panel) and posterior segment (lower panel) for four groups of mice: untreated (PBS), MSCs only, CeNP-incorporated MSCs (MSCs+CeNPs), and Au-CeNP-incorporated MSCs (MSCs+Au-CeNPs). (b) The expression of genes for IL-6, K12, and IGF-1 and the Bax/Bcl ratio in damaged corneas were assessed by real-time PCR. The percentage of F4/80^+^ macrophages in the cell suspension from the damaged eye was analyzed by flow cytometry. Quantification of NO concentration in the extract from damaged eyes (content of the eye globe in buffered saline) was analyzed by the Griess reaction. The number of PKH67^+^ cells migrated to the cornea and the histogram of the corneal opacity score of mouse groups with different treatments on day 7 after the application of treatment are also included. The number of mice in each group is *n* ≥ 6. Data were presented as means ± s.e.m. *P* values were analyzed by a two-sample *t* test, where *P < 0.05, **P < 0.01, ***P < 0.001, and ****P < 0.0001. (c) Representative images of the alkali-burned cornea after 7 days of treatment. Images represent healthy conditions, untreated conditions (intravitreal injection with PBS only), MSC injection, CeNP-incorporated MSCs (62 μg mL^–1^ of CeNPs incubated with MSCs for 24 h), and Au-CeNP-incorporated MSCs (62 μg mL^–1^ Au-CeNPs incubated with MSCs for 24 h).

To further evaluate the therapeutic effects of CeSAN-bots on corneal repair and gain deeper insights into the underlying repair mechanisms, we conducted a fluorescence microscopy analysis of frozen corneal sections. Figure 9a shows frozen sections of the cornea highlighting the corneal thickness 9 days after an alkali burn. In the healthy cornea, a normal morphology is evident, with approximately five layers of corneal epithelial cells and a stroma without swelling. In contrast, the untreated alkali-burned cornea exhibits a thinned corneal epithelium and significant stromal swelling. Notably, corneas treated with the combination of MSCs and CeNPs/Au-CeNPs displayed some epithelial thinning but maintained an epithelial morphology most closely resembling that of the healthy cornea (Figure 9b). Treatment with MSCs alone resulted in reduced stromal swelling compared to that of the untreated group (Figure 9c). Flow cytometry analysis of keratin 12-positive (K12^+^) cells in the cornea revealed a decreased proportion of K12^+^ cells in untreated, damaged corneas compared with healthy corneas. Furthermore, corneas from mice treated with the combination of MSCs and CeNPs showed a significantly higher percentage of K12^+^ cells compared to untreated corneas, indicating enhanced epithelial repair (Figure 9d). As shown in the schematic diagram in Figure 9e, the corneal response to an alkali burn involves epithelial thinning and stromal swelling, resulting from both direct chemical damage and the inflammatory response. The injury causes epithelial loss and impaired regeneration, while inflammation activates keratocytes and fibroblasts, disrupting the stromal matrix and leading to fluid accumulation. Based on the *in vitro* and *in vivo* results, we propose a mechanism for the repair process in CeSAN-bot-incorporated MSC therapy. This mechanism involves a synergistic approach where MSCs promote epithelial regeneration and stromal remodeling, while CeSAN-bots enhance these processes by reducing oxidative stress and modulating the immune response. Additionally, MSCs help reduce stromal swelling by preventing excessive fibrosis and fluid accumulation. Together, MSCs and CeSAN-bots work to restore corneal integrity, reduce swelling, and promote epithelial recovery, ultimately improving corneal transparency and function. However, this remains a proposed mechanism, and further in-depth studies are still needed to fully investigate the underlying processes and effectiveness of this therapeutic approach.

**Figure 9:**
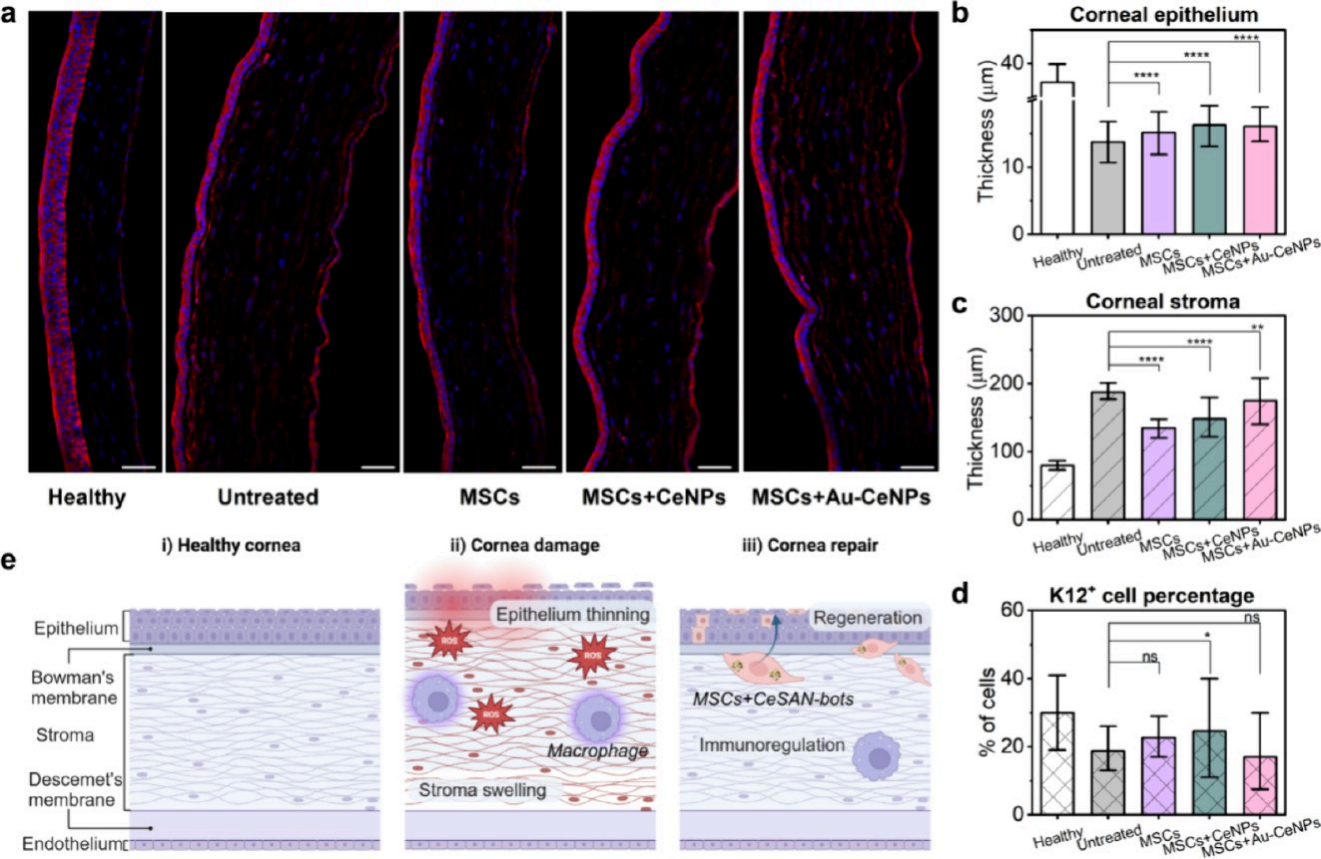
Histological evaluation of the therapeutic effect of CeSAN-bots on damaged cornea. (a) Frozen sections of the cornea 9 days after alkali burn of the corneal epithelium from healthy and untreated mice and mice treated with MSCs alone, MSCs loaded with CeNPs, or MSCs loaded with Au-CeNPs. The representative picture shows the central region of the cornea (the corneal epithelium, stroma, and endothelium) stained with DAPI (blue nuclei) and phalloidin (red cytoskeleton). The thickness of the section is 10 μm, the original magnification is 200×, and the scale bar is 50 μm. (b) Statistical analysis of the corneal epithelium thickness. Thickness data were collected over 50 areas across the whole corneal section. (c) Statistical analysis of the thickness of the corneal stroma layers. Thickness data were collected over 50 areas across the whole corneal section. (d) Percentage of keratin 12^+^ corneal epithelial cells corresponding to the level of regeneration of the damaged cornea. Data were collected from three independent experiments (each group contained at least six animals). (e) Schematic representation of the mechanisms of corneal damage and therapeutic effects of MSCs and CeSAN-bots for corneal repair.

Corneal transplant techniques face significant challenges, including limited donor tissue availability, graft rejection, and postoperative complications. Mesenchymal stem cell therapy offers a promising alternative as it enhances corneal repair through mechanisms that promote tissue regeneration and modulate immune responses. However, a primary limitation of MSC therapy is the reduced survival rate of these cells under oxidative stress. To address this, incorporating anti-inflammatory agents has been explored as a strategy to enhance the survival and efficacy of MSCs in biomedical applications. For instance, administering anti-inflammatory drugs such as celecoxib promotes stem cell survival by mitigating inflammation.^70^ Another promising approach involves protecting stem cells from oxidative damage. For example, CeO_2_ nanoparticles have been shown to protect human embryonic stem cells from oxidative stress-induced damage by reducing excessive reactive oxygen species (ROS) and preserving cell viability.^71,72^ Furthermore, the use of the stem cell secretome, which contains bioactive molecules with anti-inflammatory properties, has demonstrated improved corneal healing.^73^ In this context, the anti-inflammatory activity of CeSAN motors not only enhances MSC survival but also directly supports corneal repair and regeneration.

Although stem cell-based therapies in regenerative medicine show significant potential for repairing damaged tissues and treating degenerative diseases, their broader application is limited due to their sensitivity to the external environment, immune responses, and integration into damaged tissues. Micro/nanorobotic systems are increasingly crucial in regulating cell behaviors, with most existing applications focused on cell transport and manipulation,^74^ while intracellular delivery is gaining attention. Despite promising results in targeted drug delivery based on nanorobots, particularly to cancer cells, many systems still rely on external actuation rather than fully utilizing chemically powered micro/nanomotors.^75^ Chemically powered micro/nanorobots are still far from practical biomedical applications due to challenges such as insufficient speed for cellular entry, limited fuel availability, and the need for safer, more biocompatible intracellular fuels beyond urea and H_2_O_2_. Table S2 highlights these limitations while comparing recent advancements in chemically powered nanorobotic systems for intracellular drug delivery.^59,76–80^ For instance, Kwon et al.^59^ demonstrated a glucose-powered Au–Pt/egg-in-nest nanomotor capable of intracellular DOX delivery, with a significant amount of NPs present in the cells within 10 min. Compared with previous reports, CeSAN-bots achieve a significant two-fold increase in cellular uptake in just 1 h, proving their potential therapeutic efficacy in both *in vitro* and *in vivo* studies.

## Conclusions

In this work, we report a successful design of an enzyme-free, chemically powered, single-atom-decorated, cerium oxide-based nanoscopic robotic assembly, namely, CeSAN-bots. Powered by biocompatible fuels, such as glucose, these self-propelled CeSAN-bots demonstrate enhanced self-diffusiophoresis and long-range chemotaxis toward glucose sources. Their motility further enhances cellular uptake, enabling glucose-propelled CeSAN-bots to effectively follow glucose influx accumulation in mesenchymal stem cells, extending their capabilities to target intracellular locations within cells. The intrinsic antioxidant and immunomodulatory properties of these CeSAN-bots suggest that when combined with current mesenchymal stem cell-based therapy, they can significantly improve the efficacy of damaged corneal repair.

CeSAN-bots overcome critical limitations in existing nanoparticle- and nanorobotic-based therapies, particularly in biocompatibility, fuel reliance, and precision in intracellular delivery. Current strategies using nanoparticles, such as polymeric nanoparticles, lipid nanoparticles, and exosomes, for stem cell delivery rely primarily on passive cellular uptake. However, these approaches are hindered by limited targeting precision, susceptibility to immune clearance, and the absence of active propulsion mechanisms, reducing their overall efficacy.^81,82^ CeSAN-bots offer distinct advantages through their autonomous motion and ultrasmall size. This motion enables efficient and precise delivery to target cells and tissues, overcoming the barriers faced by traditional systems. Additionally, CeSAN-bots enhance catalytic efficiency through atomic-level heterogeneity, addressing issues such as enzyme activity loss and limited active site exposure seen in other systems. Their ability to penetrate dense tissue environments and deliver cargo precisely further positions them as a promising platform for intracellular delivery without the challenges of immune response or compromised cell viability typically associated with viral vectors and other delivery systems. Overall, these results provide strong evidence for the potential use of enzyme-free, chemically powered single-atom nanorobots as efficient therapeutic nanomedicine for next-generation precision intracellular therapy.

## Methods

### Chemicals

Poly­(acrylic acid), cerium nitrate hexahydrate (CeNO_3_)_3_·6H_2_O, 99.99%), gold­(III) chloride trihydrate (HAuCl_4_·3H_2_O, 99.9%), chloroplatinic acid hexahydrate (HPtCl_6_·6H_2_O, >37.5% Pt basis), silver nitrate (AgNO_3_, 99%), palladium­(II) nitrate dihydrate (Pd­(NO_3_)_2_·2H_2_O, >40% Pd basis), ammonium hydroxide (NH_4_OH, >25% NH_3_), sodium borohydride (NaBH_4_, 99%), iron­(III) nitrate nonahydrate (Fe­(NO_3_)_3_·9H_2_O, 99.95%), α-d-glucose (anhydrous 96%), phosphate-buffered saline (concentrated), hydrogen peroxide (H_2_O_2_, 30% w/w in H_2_O), potassium hydroxide (KOH, 90%), ethanol (CH_3_CH_2_OH, absolute), and agarose were all purchased from Sigma-Aldrich (Merck, Germany). The deionized (DI) water used in all experiments was of type I ultrapure quality (18.2 MΩ cm) from a qualified purification system.

### Synthesis

The CeNPs solutions were prepared by a wet-chemical precipitation method, as described previously.^17^ Briefly, Ce­(NO_3_)_3_·6H_2_O (2.5 mmol) and poly­(acrylic acid) (0.5 g) were mixed in DI water. Noble metal salt precursors (0.25 mmol, 1 mL of water) were added. NaBH_4_ (0.125 mmol, 1 mL of ethanol) and NH_4_OH (>25%, 15 mL) were dropwise added. A similar procedure was used to synthesize Fe-CeNPs with 2.5 mmol of Fe­(NO_3_)_3_·9H_2_O added for Fe-CeNPs synthesis. The reaction mixture was kept stirring under room temperature for 96 h followed by 10 min of centrifugation at 5580*g* (Sorvall LEGEND X1, Thermo Scientific). The supernatant was further centrifuged with an ultracentrifuge (Optima MAX-XP, Beckman Coulter) at 230,000*g* for 8 h. Colloid sediments were collected each time and washed with DI water, following extensive sonication. At least three washing cycles were repeated or until pH was neutral. Final sediments were collected and dispersed in DI water or dried at 60 °C in an oven for further characterization.

### Physicochemical Characterization

An attenuated total reflectance Fourier transform infrared (ATR-FTIR) spectrometer (Vertex 70v, Bruker, Germany) was used to characterize the polymer coatings. The crystalline structure was determined by XRD using a Rigaku SmartLab 3 kW diffractometer equipped with a Cu Kα anode X-ray tube operated at 40 kV and 30 mA. Surface chemical composition was studied by XPS using a Kratos Analytical Axis Supra instrument with a monochromatic Al Kα (1486.7 eV) excitation source. All spectra were calibrated to the adventitious C 1s peak at 284.8 eV and fitted using KolXPD (kolibrik.net). Light adsorption was measured by using a Jasco V-750 UV–visible absorption spectrophotometer. Hydrodynamic diameter and zeta potential measurements were performed in water and PBS solutions (pH 7.4) using a Malvern Panalytical Zetasizer Ultra instrument.

### Transmission Electron Microscopy

The TEM samples were prepared from dispersed CeNPs in aqueous solutions (1 mg mL^–1^) drop-cast on lacy carbon-covered copper TEM grids coated by an ultrathin carbon membrane (Agar Scientific, UK). TEM was conducted with a microscope TITAN Themis 60-300 (Thermo Fisher Scientific, USA) equipped with a high-brightness X-FEG Schottky electron emitter but without a *C*_*s*_ probe spherical aberration corrector. High-resolution STEM imaging was performed at an acceleration voltage of 300 kV and with a beam current of ∼30 pA, which resulted in a lateral image resolution of ∼1.3 Å. The detection of Au single atoms via HR-STEM was achieved with atomic *Z*-contrast conditions determined by the inner collection angle of the used HAADF detector, which was higher than 70 mrad. Note the example of a medium-angle ADF (MAADF) image with the inner angle of 21 mrad (Figure S5), which suggests that this imaging condition with a substantially increased signal-to-noise ratio is still capable of detecting single Au atoms at CeNPs while it can be compared with a simultaneously acquired HAADF image. The presented images were acquired and processed with Velox v2.14 software, and no additional inverse fast Fourier transformation filters were applied.

STEM-EELS elemental mapping and spectroscopy were performed at an acceleration voltage of 300 kV (to achieve the highest lateral image resolution) with a Gatan Image Filter Quantum 966/ERS spectrometer equipped with an UltraScan1000 CCD detection camera. EELS acquisition and processing were performed in Gatan Microscopy Suite v3.3 software. The EEL spectral data-cube (Figure 2e,f) was acquired in DualEELS mode in the form of simultaneously collected low-loss spectrum images with a zero-loss peak and core-loss spectrum images with Ce-M_5_ and Au-M_5_ edges lying at 883 and 2206 eV, respectively. To yield a detectable signal from the Au edge at very high energy without substantial electron beam damage to the CeNPs structure, the acquisition conditions had to be optimized with the following parameters: the probe current was ∼100 pA; convergence and collection semiangles were 10 and 36 mrad, respectively; the dispersion was 1 eV, with a channel giving energy readout range of 757–2803 eV; detector binning was [1, 130]×; the pixel size and pixel total time were 0.35 nm and 0.1 s, respectively; the number of acquired frames was 1; HQ dark correction was applied. Afterward, the Ce-M and Au-M EELS edge signal maps were obtained with the following setup: plural scattering in core-loss spectra was removed via Fourier deconvolution using the low-loss spectra with a zero-loss peak, the background subtraction model was a first-order log-polynomial, ELNES was excluded, and the cross-sectional model was Hartree–Slater.

STEM-EDXS elemental mapping and spectroscopy were performed at the acceleration voltage of 60 kV (to mitigate the electron beam damage to the original CeNPs structure during long acquisition times) with a Super-XG1 spectrometer containing four 30 mm^2^ windowless detectors, giving a solid detection angle of ∼0.7 sr. The EDXS data were acquired and processed in Velox v2.14 software. The used STEM-EDXS elemental mapping acquisition conditions (Figure 2g,h) were optimized with the following parameters: the probe current was ∼100 pA; dispersion was 5 eV, with a channel giving energy readout range of 20 keV; the pixel size and pixel dwell time were 0.09 nm and 60 μs, respectively; the image size was 152 × 293 pixels; the number of acquired frames was 578; the total acquisition time was 31 min. The net intensity maps of Ce-L and Au-L lines were created with the use of a maximum likelihood fit method for the deconvolution of overlapping peaks and an empirical model for spectrum background subtraction. The standard Cliff–Lorimer (*K*-factor) method (embedded in the software) was used for the quantification of the chemical composition to the atomic percentage concentrations from the EDX spectra integrated from the volume of the mapped Au-CeNPs structure.

### Catalytic Activity of Glucose Oxidation

The catalytic activity of CeNPs and M-CeNPs was characterized by incubation of glucose (0.5 mg mL^–1^) with NPs (0.5 mg mL^–1^) for 20 min followed by quantifying the unreacted glucose using a glucose (HK) assay kit (GAHK20, Sigma-Aldrich, Germany). The catalase-mimicking activity of NPs disproportioning H_2_O_2_ was measured by a catalase assay kit (MAK381, Sigma-Aldrich, Germany). The coupled cascade GOx and CAT mimicking activities of NPs were monitored by measuring the UV–visible absorption spectra at 290 and 400 nm.

### Electrochemical Tests

Glucose electro-oxidation cyclic voltammetry of Au-CeNPs was measured in 0.1 M KOH at room temperature. Ten μL of NPs mixed with Nafion solution (final ratio 40:1) was dried on the glassy carbon electrode serving as the working electrode. A Pt wire served as the counter electrode, and the Ag/AgCl (1 M KCl) electrode was used as the reference electrode. Before the electrochemical test, the electrolyte solutions were purged with either ambient air (21% O_2_) or N_2_ for at least 30 min. The cyclic voltammograms were recorded by applying proper potential ranges at a scan rate of 10 mV/s using an Autolab potentiostat (Metrohom, Switzerland).

### Motion Behavior

DLS was conducted by using a Malvern Panalytical Zetasizer Ultra instrument. A colloidal solution of CeNPs and M-CeNPs (0.025 mg mL^–1^, 1 mL) in PBS solutions was used in addition to different concentrations of glucose (1 to 20 mg mL^–1^). The backscattering data with a scattering angle of 173° were collected as intensity-based scattering and transformed into a diffusion coefficient and relaxation time based on the Malvern software ZS explorer (V1.3.2.27). For the long-range chemotaxis setup, a Petri dish with a 3.5 cm diameter was filled with 5 mL of PBS (pH 7.4) solutions. A 1% agarose gel cylinder 0.6 mm in diameter and 0.5 mm in length was presoaked in 1 M glucose solutions overnight. The agarose gel cylinder was placed at the edge of the Petri dish. After placing the cylinder, the Petri dish was kept still without perturbation for 60 min to allow sufficient glucose diffusion. Different CeNPs and M-CeNPs (20 mg mL^–1^) were introduced at the central point of the Petri dish using a needle syringe. Ten μL of samples was withdrawn at different locations and different time points. The collected samples were analyzed by a UV–visible absorption spectrometer and subsequently converted to concentration based on CeNPs absorbance at 290 nm.

### *In Vitro* Tests

For fluorescently tagged NPs, DiI (1 mg mL^–1^ in dimethyl sulfoxide, Thermo Fisher Scientific) was added to NPs (1 mg mL^–1^ in water) with a volume ratio of 1:100, followed by 8 h of dialysis with a 10K MWKO membrane (Thermo Scientific). Concentrations of both DiI and CeNPs were calibrated by UV–visible spectrometry, where the absorptivity coefficient of CeNPs at 288 nm was around 25.2 L g^–1^ cm^–1^ (Note S1), and DiI at 556 nm was around 76 L g cm^–1^. Detailed *in vitro* experimental procedures for MSC preparation and other steps are described in Note S9. The metabolic activity of MSCs was determined by a water-soluble tetrazolium-1 (WST-1) assay (Roche, Mannheim, Germany). MSCs were labeled with a fluorescent dye PKH67 green fluorescent cell linker kit (Sigma-Aldrich) to monitor their fate after intravitreal administration. The fluorescence intensity and homogeneity of the staining were tested by flow cytometry (LSRII cytometer, BD Biosciences, Franklin Lakes, New Jersey, USA) and fluorescence microscopy (Microscope Axioskop, Zeiss, Oberkochen, Germany). To confirm the uptake and localization of NPs@DiI to PKH67-labeled MSCs, fluorescent microscopy was performed. MSCs were stained with PKH67, seeded on circular cover glasses (3.5 × 10^4^/glass), placed in a 24-well culture plate (TPP), and cultured in 200 μL of complete DMEM for 24 h at 37 °C in a humidified atmosphere of 5% CO_2_ to allow MSCs to adhere before the addition of NPs. After 24 h, NPs in the concentration of 62 μg mL^–1^ were added to wells with MSCs and cultured for 90 min in a total volume of 300 μL of complete DMEM. Subsequently, cover glasses with grown MSCs were fixed with 4% formaldehyde for 1 h (Lachema, Brno, Czech Republic) and mounted in a Vectashield mounting medium with DAPI (Vector Laboratories, Newark, California, USA). Samples were analyzed using a fluorescence microscope Axioskop (Zeiss) and processed by software Isis (MetaSystems, Heidelberg, Germany). For analysis of NP internalization, MSCs (4 × 10^5^/well) were cultured in a 6-well plate (TPP) in a total volume of 2 mL of complete DMEM to allow MSCs to adhere for 24 h before the addition of NPs. Subsequently, after NP addition (100 μg mL^–1^), NP+MSCs were sampled at five different time points (to NP incubation with MSCs for durations of 0.5, 1, 3, 6, and 24 h). Control MSCs cultured without NPs were also included. Then, MSCs were washed with PBS and harvested with 0.5% trypsin for 5 min, followed by gentle scraping and dilution in PBS. Hoechst 33258 (Sigma-Aldrich) was added to all samples before testing for staining of dead cells. Data were collected using a flow cytometer LSRII (BD Biosciences) and analyzed using FlowJo software (BD). Analysis of the NP influence on MSC cytokine production and gene expression was carried out by ELISA and real-time PCR. Briefly, MSCs (8 × 10^4^/well) were cultured for 24 h in a 24-well plate (TPP) in a total volume of 500 μL of complete DMEM to allow MSCs to adhere before NP addition. After 24 h, selected concentrations of NPs (31, 62, and 125 μg mL^–1^) were added, and MSCs were cultured with NPs in a total volume of 700 μL for an additional 24 h. Control MSCs cultured without NPs were also included. After 48 h, culture supernatants were harvested and MSCs were collected to a TRI reagent (Molecular Research Center, Cincinnati, Ohio, USA) and stored at −80 °C. The production of hepatocyte growth factor (HGF) and interleukin-6 (IL-6) was quantified in supernatants collected from NP-exposed MSC cultures by DuoSet ELISA kits (R&D Systems, Minneapolis, Minnesota, USA) according to the manufacturer's instructions. Optical density was measured using a Sunrise spectrophotometer (Tecan, Männedorf, Switzerland) and analyzed by Kim 32 software (Schoeller Instruments, Prague, Czech Republic), and according to cytokine standards of known concentration, concentration in pg mL^–1^ was calculated.

To determine the effect of NPs on glucose metabolism of MSCs, 1 × 10^4^ MSCs/well were cultured in 96-well tissue culture plates (Nunc, Roskilde, Denmark) in 50 μL of complete DMEM for 6 h at 37 °C in a humidified atmosphere of 5% CO_2_. After this period, 62 μg mL^–1^ CeNPs and Au-CeNPs diluted in complete DMEM were added to wells of 50 μL volume, and MSCs were cultured with NPs in a total volume of 100 μL of complete DMEM. Control MSCs cultured without NPs were also included. The metabolic activity of MSCs was assessed by the WST-1 assay. Changes in expression of genes for PDH and HIF-1α were tested by real-time PCR.

ROS determination *in vitro* was performed according to a previously established procedure.^17^ MSCs (3 × 10^5^/well) were cultured in a 6-well plate for 24 h alone to adhere before addition of NPs; then, 62 μg mL^–1^ CeNPs or Au-CeNPs were added to MSCs for an additional 24 h. MSCs were washed with PBS and incubated with 50 μM 2′,7′-dichlorofluorescin diacetate (DCDHF-DA, Sigma-Aldrich) diluted in DMEM without FBS for 50 min at 37 °C. MSCs were then harvested, washed with PBS, and incubated alone or with 500 μM H_2_O_2_ for 30 min at room temperature to induce oxidative stress of MSCs. The intensity of the DCDHF-DA fluorescence signal (emission of 530 nm) was assessed by a flow cytometer LSRII.

Macrophages were obtained from the peritoneal lavage of three mice, and 1.1 × 10^6^ of peritoneal cells per well were cultured in a 24-well plate in a total volume of 750 μL of RPMI 1640 medium (Sigma-Aldrich) supplemented with 10% fetal bovine serum, antibiotics (100 U/mL penicillin, 100 μg mL^–1^ streptomycin), and 10 mM HEPES buffer (referred to as complete RPMI) for 72 h. Macrophages were cultured unstimulated or stimulated with 10 μg mL^–1^ lipopolysaccharide (Sigma-Aldrich) and 10 ng mL^–1^ interferon-γ (PeproTech) and cultured with selected concentrations of CeSAN-bots. The concentration of NO was measured in culture supernatants collected after 72 h of cultivation by the Griess reaction. A mixture of 50 μL of 1% sulfanilamide and 50 μL of 0.3% *N*-1-naphthyl ethylenediamine dihydrochloride (both in 3% H_3_PO_4_) was incubated with 100 μL of the tested culture supernatant. Nitrite was quantified by spectrophotometry at 540 nm using sodium nitrite as a standard. For flow cytometry, activated and control macrophages were harvested after 72 h of cultivation, transferred to PBS, and incubated for 30 min at 4 °C with an antimouse allophycocyanin-labeled anti-CD80 (clone 16-10A1) monoclonal antibody purchased from BioLegend. Hoechst 33258 was added to all samples before testing for the staining of dead cells. Samples were then analyzed using an LSRII flow cytometer, and the percentage of CD80^+^ macrophages was determined using FlowJo software.

For the analysis of T-cell activation and proliferation by flow cytometry, the spleen of the BALB/c mouse was cut into small pieces, homogenized, and filtered to prepare a single-cell suspension. One million spleen cells per well were cultured in a 48-well plate (TPP) in a final volume of 1 mL of complete RPMI for 72 h unstimulated or stimulated with 1 μg mL^–1^ concanavalin A (Sigma-Aldrich). After 72 h, spleen cells were harvested, transferred to PBS, and incubated for 30 min at 4 °C with antimouse allophycocyanin-labeled anti-CD3 (clone 17A2) and fluorescein isothiocyanate-labeled anti-CD25 (clone PC61) monoclonal antibodies purchased from BioLegend. Hoechst 33258 was added to all samples before testing for staining of dead cells. Samples were then analyzed using an LSRII flow cytometer, and the percentage of CD3^+^CD25^+^ T-lymphocytes was determined using FlowJo software.

To assess T-lymphocyte proliferation, isolated T cells were labeled by a CellTrace CFSE (carboxyfluorescein succinimidyl ester) cell proliferation kit according to the manufacturer's instructions. One million CFSE-labeled spleen cells per well were cultured in a 48-well plate (TPP) in a final volume of 1 mL of complete RPMI for 96 h unstimulated or stimulated with 1 μg mL^–1^ concanavalin A (Sigma-Aldrich). After 96 h, spleen cells were harvested and transferred to PBS with Hoechst 33258 to stain dead cells. Samples were then analyzed using an LSRII flow cytometer, and the percentage of highly proliferating (CFSE^low^) T-lymphocytes was determined using FlowJo software.

### *In Vivo* Tests

Female BALB/c mice at the age of 8–14 weeks were obtained from Envigo Company (Indianapolis, Indiana, USA). The use of animals was approved by the Local Ethical Committee of the Institute of Experimental Medicine of the Czech Academy of Sciences, Prague (approval number 7448/2023). For establishing a corneal damage model, BALB/c mice were anesthetized using an intraperitoneal injection of a mixture of xylazine (Bioveta, Ivanovice, Czech Republic) and ketamine (Bioveta). The surface (corneal and limbal region) of the left eye was damaged by the application of 3 mm-diameter filter paper soaked with 3 μL of 0.25 M NaOH for 30 s. The eye was then thoroughly rinsed with 10 mL of PBS. Before intravitreal application, MSCs (3 × 10^5^/well) or PKH67-labeled MSCs (for the migratory assay) were cultured in a 6-well plate (TPP) in a total volume of 2.5 mL of complete DMEM to allow MSCs to adhere for 24 h before addition of NPs. Subsequently, NPs (62 μg mL^–1^) were added to MSCs to incubate for another 24 h. Control MSCs cultured without NPs were also included. After 48 h, MSCs were washed with PBS and harvested by 0.5% trypsin for 5 min and gentle scraping, centrifuged (250*g* for 8 min), and diluted in PBS (5 × 10^7^/mL). Forty-eight hours after corneal damage, mice were anesthetized using an intraperitoneal injection of a mixture of xylazine (Bioveta) and ketamine (Bioveta). The intravitreal application of MSCs was performed by a Hamilton syringe with a maximal volume of 5 μL (Hamilton, Reno, Nevada, USA) with a 33G needle (Hamilton). Mice were divided into four groups and received 1 μL of treatment to the left damaged eye (the untreated group received 1 μL of PBS). Each group contained at least six animals. After 7 days, mice were sacrificed, and eyeballs were enucleated and processed for the next analysis.

For the migratory assay, the left eyeballs were enucleated 7 days after the intravitreal application of the treatment. Eyeball samples were divided into the anterior segment (cornea and limbus) and posterior segment using a Leica Wild M651 microsurgical microscope (Leica). These segments were then cut into pieces and digested with 1 mg mL^–1^ collagenase I (Sigma-Aldrich) in HBSS for 50 min at 37 °C to prepare a single-cell suspension. Hoechst 33258 (Sigma-Aldrich) was added to all samples before testing for staining of dead cells. Cell suspensions from anterior and posterior segments were then analyzed using an LSRII flow cytometer (BD Biosciences), and the number of PKH67^+^ cells and DiI^+^ cells was determined using FlowJo software (BD). The corneal opacity was scored 9 days after cornea alkali burn (7 days after treatment) using the microsurgical microscope. Scoring was performed according to a previous report.^69^ Opacity level 0 means a clear cornea with no opacity, opacity level 1 means minimal haze and no opacity, opacity level 2 means mild or district opacity of the damaged cornea, opacity level 3 means moderately dense opacity with partially visible intraocular structures, and opacity level 4 means severe opacity with no visible intraocular structures.

Damaged corneas were extracted using a microsurgical microscope, collected to TRI reagent (Molecular Research Center), and stored at −80 °C until their analysis using real-time PCR. Healthy corneas from control mice were extracted, proceeded the same way, and used as a healthy control. After extraction of corneas, the rest of the eyes were centrifuged (250*g*, 8 min) in 400 μL of HBSS and utilized for the preparation of extracts, which were analyzed by the NO assay. Total RNA was extracted from both *in vitro* tested NP-exposed MSCs and corneas were extracted using a TRI reagent (Molecular Research Center) according to the manufacturer′s instructions. One microgram of total RNA was treated with deoxyribonuclease I (Promega, Madison, Wisconsin, USA) and used for subsequent reverse transcription. The first-strand cDNA was synthesized with random hexamers (Promega) in a total reaction volume of 25 μL using M-MLV reverse transcriptase (Promega). Quantitative real-time PCR was performed using a Power SYBR Green PCR Master Mix (Applied Biosystems, Foster City, California, USA) on a cycler StepOnePlus real-time PCR system (Applied Biosystems) with parameters including denaturation at 95 °C (3 min) followed by 45 cycles at 95 °C (20 s), annealing at 60 °C (30 s), and elongation at 72 °C (30 s). Fluorescence data were collected at each cycle after the elongation at 80 °C for 5 s. Collected data were analyzed by StepOne software version 2.3 (Applied Biosystems). For calculation of the relative expression of the analyzed gene, it was compared with the glyceraldehyde 3-phosphate dehydrogenase (GAPDH) gene. The primers (all from Generi Biotech, Hradec Kralove, Czech Republic) used for amplification are shown in Table S3. The concentration of nitric oxide (NO) was measured in extracts from healthy and damaged eyes by the Griess reaction.

For the flow cytometry analysis of infiltration of the damaged eye by macrophages, single-cell suspensions prepared from control and damaged eyes using collagenase I (50 min, 37 °C) were washed in PBS and incubated for 30 min at 4 °C with an antimouse allophycocyanin-labeled anti-F4/80 (clone BM8) monoclonal antibody purchased from BioLegend (San Diego, California, USA). Hoechst 33258 (Sigma-Aldrich) was added to all samples before testing for staining of dead cells. Samples were then analyzed using an LSRII flow cytometer (BD Biosciences), and the number of F4/80^+^ macrophages was determined using FlowJo software (BD).

For the histological analysis, 9 days after the alkali burn of the cornea, eyeballs were enucleated and fixed in 4% paraformaldehyde (Sigma-Aldrich) for 1 h, followed by overnight cryoprotection in 15% sucrose (Sigma-Aldrich). The eyes were embedded in a Tissue-Tek O.C.T. Compound (Sakura Finetek), and frozen sections at a thickness of 10 μm were prepared using a Cryostat Leica CM1950. The sections were fixed with 4% paraformaldehyde for 10 min, washed in PBS, and mounted with a mixture of Vectashield antifade mounting medium with DAPI (Vector Laboratories) and a Vectashield antifade mounting medium with phalloidin (Vector Laboratories). Mounted sections were analyzed by a confocal fluorescence microscope Andor BC43 (Andor Technology).

Seven days after the intravitreal application of the treatment, corneal regeneration was analyzed using flow cytometry. Corneas were extracted from eyeballs using a microsurgical microscope, cut into pieces, and digested with 1 mg mL^–1^ collagenase I in HBSS for 50 min at 37 °C to prepare a single-cell suspension. Cells were then stained with a Zombie Violet viability kit (BioLegend), fixed and permeabilized using IC fixation and permeabilization buffers (eBioscience), and stained for 30 min at 4 °C by an anti-K12 monoclonal antibody conjugated with Alexa Fluor 488 (Santa Cruz Biotechnology) to analyze the K12^+^ cell percentage.

## Supplementary Material



## Data Availability

The authors declare that all data supporting the findings of this study are available within the article and its Supporting Information. Any additional requests for information can be directed to the corresponding author.

## Notes

Additional data (figures and tables) and notes about CeSAN-bot characterization by FTIR spectra, HR-STEM images, UV–vis spectra, XPS spectra, HAADF-STEM images, EDX mapping, electrochemical switching potential curves on glucose oxidation, DLS, propulsion speed at different ironic strength solutions, glucose diffusion and prebalance time map, CeSAN-bots' influence on MSC cell metabolism histogram, comparison of glucose-powered nanobots and chemically powered nanobots for intracellular delivery, and murine primer sequences used for RT-PCR (PDF)
