# Supplementary material for: Single-Atom Colloidal Nanorobotics Enhanced Stem Cell Therapy for Corneal Injury Repair
Source: ACS Nano. 2025 May 13;19(20):19095–115. doi: 10.1021/acsnano.4c18874 (PMC12120985; doi:10.1021/acsnano.4c18874)
Supplement: Supplementary file 1 [file nn4c18874_si_001.pdf]

Supplementary Information for

Single-Atom Colloidal Nanorobotics Enhanced Stem Cell Therapy for Corneal Injury Repair

Xiaohui Ju[1], Eliška Javorková[2,3], Jan Michalička[4], Martin Pumera [1,5,6]*

[1]Future Energy and Innovation Laboratory, Central European Institute of Technology, Brno University of Technology, Purkyňova 123, Brno, 61200, Czech Republic

[2]Department of Toxicology and Molecular Epidemiology, Institute of Experimental Medicine, Academy of Sciences of the Czech Republic, Vídeňská 1083, Prague, 14200, Czech Republic

[3]Department of Cell Biology, Faculty of Science, Charles University, Viničná 7, Prague, 12844, Czech Republic

[4]Central European Institute of Technology, Brno University of Technology, Purkyňova 123, Brno, 61200, Czech Republic

[5]Advanced Nanorobots & Multiscale Robotics Laboratory, Faculty of Electrical Engineering and Computer Science, VSB - Technical University of Ostrava, 17. listopadu 2172/15, 70800 Ostrava, Czech Republic

[6]Department of Medical Research, China Medical University Hospital, China Medical University, No. 91 Hsueh-Shih Road, Taichung 40402, Taiwan

Table of Contents

Supplementary Notes 3

Supplementary **Note S1.** UV-visible spectrophotometry for CeNPs quantification..........3

Supplementary **Note S2.** Quantification of glucose oxidation with glucose assay kit 4

Supplementary **Note S3.** Electrochemical measurement for glucose oxidation mechanism 5

Supplementary **Note S4.** Diffusion parameters calculation based on Stokes equations..........7

Supplementary **Note S5.** The hydrodynamic diameter measured by DLS 8

Supplementary **Note S6.** Python codes for glucose diffusion simulation..........10

Supplementary **Note S7.** Notes about the chemotaxis of glucose powered nanorobots..........12

Supplementary **Note S8.** Detailed experimental procedures for *in vitro* experiments 14

Supplementary Figures 16

Supplementary **Figure S1.** ATR-FTIR characterization 16

Supplementary **Figure S2.** HR-STEM images of CeSAN-bots 17

Supplementary **Figure S3.** UV-visible absorption spectra and bandgap calculation 18

Supplementary **Figure S4.** XPS Ce 3*d* core-level spectra..........19

Supplementary **Figure S5.** HAADF-STEM images identifying Au single atoms on Au-CeNPs..........20

Supplementary **Figure S6.** EDX spectra and elemental composition of Au-CeNPs..........21

Supplementary **Figure S7.** The switching potential effect on glucose oxidation..........22

Supplementary **Figure S8.** Scheme of possible adsorption species on the Au-CeO_2 surface 23

Supplementary **Figure S9.** Hydrodynamic diameter by DLS 24

Supplementary **Figure S10.** Propulsion speed in different ionic strength solutions 25

Supplementary **Figure S11.** Glucose diffusion and the effect of pre-balance time..........26

Supplementary **Figure S12**. CeSAN-bots influence on the cell metabolism of MSCs..........27

Supplementary **Figure S13**. General flow cytometry gating strategy. 28

Supplementary **Table S1**. Comparison of glucose-powered nanorobots..........29

Supplementary **Table S2**. Comparison of chemically-powered nanorobots for intracellular delivery ...30

Supplementary **Table S3**. Murine primer sequences used for real-time PCR..........31

References..........32

Supplementary Notes

Supplementary Note S1. UV-visible spectrophotometry for CeNPs quantification

The absorbance of CeNPs and M-CeNPs aqueous dispersions (where M-CeNPs refer to Au-CeNPs, Pt-CeNPs, Ag-CeNPs, and Pd-CeNPs) were measured with a UV-visible absorption spectrophotometer (JASCO, V-750). CeNPs absorption spectra were recorded in the range λ = 190 – 700 nm at room temperature (T = 25 °C). According to the Beer-Lambert law, $A_{CeNPs}(\lambda)$ is proportional to the concentration of CeNPs c_{CeNPs}: $A_{CeNPs}(\lambda) = \varepsilon_{CeNPs}(\lambda) \times l \times c_{CeNPs}$, where $\varepsilon_{CeNPs}(\lambda)$ are the absorptivity coefficient and l the cell thickness. Given l = 1 cm for the standard quartz SUPRASIL cell (Hellma, QS.10) and ε_{CeNPs} = 25.2 L. g^{-1} cm^{-1} at the characteristic ~ 288 nm peak[1-3], the concentration of all synthesized CeNPs was evaluated using the Beer-Lambert law with an accuracy better than 0.1%.

All UV-visible absorption spectra were treated in the same way: (i) subtract the UV-visible absorption spectrum of the solvent ($A_{H_2O}(\lambda)$) and (ii) normalize by the absorbance of CeNPs at λ = 288 nm ($A_{CeNPs}(\lambda = 288\ nm)$), according to the following equation:

$$\tilde{A}_{CeNPs}(\lambda) = \frac{A_{CeNPs}(\lambda) - A_{H_2O}(\lambda)}{A_{CeNPs}(\lambda = 288\ nm)} \qquad (1)$$

Supplementary Note S2. Quantification of glucose oxidation with glucose assay kit

In most glucose assays, the quantification of glucose oxidase (GOx) activity is based on indirect quantification of H_2O_2. This is because GOx can convert glucose to gluconic acid and H_2O_2, where the latter can be precisely quantified based on colorimetric methods applying UV-visible absorbance spectrophotometry. Such quantification has been applied in previously reported research[4,5], commercially developed glucose quantification kits (for example, GAGO20 from Sigma Aldrich), and most developed glucose sensors[6] (for measuring blood glucose level). However, in the context of currently developed materials, CeNPs are known for their robust catalase-mimicking activity to disproportionate H_2O_2 comparable to natural catalase. We cannot simply rely on indirect H_2O_2 quantification for M-CeNPs glucose catalytic activities, as with most of the reported methods. Therefore, we have applied the glucose quantification kit (Glucose (HK) Assay Kit, MAK263, Sigma Aldrich) for subsequent glucose quantification in this work.

The general principle of quantification is as follows:

$$glucose + ATP \xrightarrow{hexokinase} \text{glucose-6-phosphate (G6P)} + \text{ADP} \quad (2)$$

$$G6P + NAD \xrightarrow{G6PDH} \text{6-phospho-gluconate (G6PDH)} + \text{NADH} \quad (3)$$

In the assay, glucose is phosphorylated by adenosine triphosphate (ATP) in the reaction catalyzed by hexokinase. Glucose-6-phosphate (G6P) is then oxidized to 6-phosphogluconate in the presence of oxidized nicotinamide adenine dinucleotide (NAD), in a reaction catalyzed by glucose-6-phosphate dehydrogenase (G6PDH). During this oxidation, an equimolar amount of NAD is reduced to NADH. The consequent increase in absorbance at 340 nm is directly proportional to glucose concentration. In this way, the effect of H_2O_2 can be eliminated.

Supplementary Note S3. Electrochemical measurement for glucose oxidation mechanism

The electro-oxidation of glucose catalyzed by Au-CeNPs follows a proposed mechanism based on previous studies of Au-catalyzed glucose oxidation by Hsiao et al.[7] and Vassilyev et al.[8] The mechanism involves the adsorption of glucose on the electrode surface, followed by the oxidation of hydrogen atoms bound to the carbon C1 atoms. The overall reaction involves the transfer of two electrons per glucose molecule. It has been suggested that the formation of $(OH)_{ads}$ (adsorbed hydroxyl) on the gold surface is the initial step for the electrochemical oxidation of glucose. The interaction between the hemiacetal group of glucose and $(OH)_{ads}$ plays a crucial role in the oxidation process. In the case of Au-CeNPs, where strong metal support interaction (SMSI) occurs, the oxidation peak of Au to $Au(OH)_{ads}$ is not observed (this can be due to the OH already incorporated into the CeO_x structure in aqueous solutions, or the oxidation peak is too small to be observed in the absence of glucose). The hydrogen bound to the C1 carbon of glucose is first oxidized, leading to the formation of radical species. This step is rate-determining and involves the transfer of one electron to the electrode. The radical species are further oxidized to form gluconolactone, accompanied by the transfer of another electron to the electrode. Gluconolactone is then desorbed from the electrode and hydrolyzed to form gluconate.

The effect of the oxidation peak on the backward cathodic scan was further investigated by increasing stepwise the switching potential in anodic scans (E_+). As shown in **Figure S7a**, when increasing E_+, the intensity of the glucose oxidation peak decreased, while the glucose oxidation potential (E_f-glucose) remained unchanged. The decrease in intensity at higher switching potentials can be attributed to competitive chemisorption and incorporation of these chemisorbed species (most likely OH-related species)[9] into the Au-CeNPs surface. This incorporation can lead to the irreversible formation of surface oxide/oxyhydroxide species at higher cycling potentials (poisoning effect), which hinders the glucose oxidation process[9]. For the backward cathodic scan at higher E_+ values, the peak potential (E_b) shifts towards more negative values in the presence of glucose (**Figure S7b**). This shift indicates that the reduction profiles are also affected, suggesting that strongly adsorbed anions are incorporated within the oxide structure and not entirely desorbed

as the oxide/oxyhydroxide layer develops with increasing switching potential. This phenomenon is consistent with previous observations[9] related to noble metal alcohol oxidation, which also involves tolerance of competitive adsorbed species. The irreversible chemisorption/catalyst "poisoning effect" at higher potential can be further proved by scanning CV from the highest switching potential to the lowest switching potential (**Figure S7d**). An opposite trend of glucose oxidation was observed where the intensity at the E_f-glucose reached maximum during the highest switching potential at the beginning, indicating that the deactivation appears more severely at the highest potential, and it is not reversible when lowering the switching potential. We further note that following Conway's theory[9], the irreversible behavior is partly kinetic in origin and the oxide/hydroxide transformation is intimately connected as a function both of time and potential of the electrode.

In the absence of reactants (such as glucose or alcohol), the oxidation peak at the cathodic scan has not been observed on the noble metal electrode[7], thus the observed current increase is likely to be associated with Au-CeO_2 interaction with chemisorbed species. The possibility of Au-CeO_2 interaction with chemisorbed species has been clarified by performing the same experiment using only CeNPs in the absence of glucose under deoxygenated conditions (**Figure S7c**). This further indicates that the origin of the oxidation peak during the cathodic scan without glucose is most likely from the adsorbed species incorporation into Au-CeO_2 structures. However, the tendency of E_b shifts with the change of switching potential is opposite in the absence of glucose compared with that in the presence of glucose. In the case of glucose absence, more positive E_+ values induced a more positive shift in E_b. The underlying reasons for this peak and its interesting behavior in the absence of glucose are likely associated with the intrinsic properties of the Au-CeNPs interaction and the chemisorbed species under alkaline electrolyte conditions (**Figure S8**), which are beyond the scope of the current work.

Supplementary Note S4. Diffusion parameters calculation based on Stokes equations

A spherical particle in solution simultaneously undergoes translational and rotational diffusion, and its translational diffusion coefficient (D_0) and rotational diffusion coefficient (D_r), and rotational relaxation time (τ_r) can be described by the Stokes-Einstein equations (4) and (5):

$$D_0 = \frac{k_B \times T}{6 \times \pi \times \square \times R} \quad (4)$$

$$D_r = \frac{k_B \times T}{8 \times \pi \times \square \times R^3} = \tau_r^{-1} \quad (5)$$

T is the temperature, $\square$ is the viscosity of the solution, R is the effective hydrodynamic radius of the particle and τ_r is the characteristic rotational relaxation time. The equations (4) and (5) are applied to the M-CeNPs systems to derive diffusion parameters reported in the current research.

For the calculation of speed, the following equation (6) is used as proposed by Lee et al.[10]:

$$D_{app} = D_0 + \frac{l^2}{4 \times \tau_r} \quad (6)$$

where D_{app} is the apparent diffusion constant, l is the average ballistic persistence length, and τ_r is the rotational relaxation time. By using Equation (6), we can derive the average speed of the CeSAN-bots in the presence of various concentrations of glucose solutions.

Supplementary Note S5. Hydrodynamic diameter measured by DLS

Dynamic light scattering (DLS) is based on the intensity of light scattered, where the scattering intensity is biased towards larger particles. In the Rayleigh scattering regime, the intensity is proportional to the diameter of the sixth power. Therefore, even a very small amount of a larger aggregate species would have a large bias on the signal.

The intensity/volume/number distributions for Au-CeNPs in the current study were compared as shown in **Figure S9**. The somewhat larger-than-expected hydrodynamic diameters based on intensity are due to the contribution of a few larger fractions of agglomerates in the samples. Although only present in a small proportion, these large fractions will contribute more to the scattering signals. The scattering contribution from a small number of larger particles can be filtered by analyzing the data based on the number-based distribution, which gives a hydrodynamic diameter of ~9 nm for Au-CeNPs.

The particles showed a peak at a larger size for the intensity distribution and a peak at smaller sizes around 9 nm. The peak of larger sizes for the intensity distribution gradually minimized upon converting the size to a volume distribution, and finally disappeared when expressed as a number distribution. The conversion to the number distribution was done through the algorithm built into the software from Malvern instruments (Dynamic light scattering software ZS Xplorer V1.3.2.27), which also accounts for Mie absorption and scattering contributions. The refractive indices and absorption of CeO_2 at the laser wavelength of 660 nm are 2.20 and 0.06, respectively.

It should be noted that the size was not very sensitive to the value used for the refractive indices. All sizes reported in the current study are the number average sizes determined as described above as suggested by the instrumental manufacturer (Malvern Panalytical Technical note: Intensity-volume–number: which size is correct?). We believe that presenting the smaller NPs less than 10 nm in a number-weighted D_H is more representative of their true hydrodynamic diameters.

On the other hand, for the calculation of the diffusion coefficients, the rotational diffusion relaxation time of Au-CeNPs is 44 ± 6 μs, and the translational diffusion constant is 11.2 $\mu m^2.s^{-1}$.

These values are in the range for the theoretically calculated rotational relaxation time for a 40 nm sphere based on the Einstein-Stokes equations, corresponding to the measured intensity-based hydrodynamic diameter in the size of 40 nm rather than their number-based hydrodynamic diameter. Although the radii of the calculated number-based hydrodynamic diameter in the manuscript match well with XRD and TEM analysis, the data based on scattering volume and numbers are more susceptible to measurement errors. Thus, the subsequent DLS analysis of diffusion-related parameters is reported based on scattering intensity data.

Supplementary Note S6. Python codes for glucose diffusion simulation

The following code is used to simulate glucose diffusion in a circular domain (3.5 cm in diameter) using the finite difference method as shown in **Figure S11a** and **Figure 4i** (for the glucose gradient map). The glucose diffusion coefficient, temperature, viscosity of water, and the Boltzmann constant are defined as constants. The diffusion equation is discretized and solved iteratively over time, with reflective boundary conditions applied to the edges of the domain. The resulting concentration matrix is saved as an XYZ file and plotted in a logarithmic scale in 2D and 3D. The center top point of the circular domain is set as the point source. Simulation is carried out using open-source software Pycharm 2023.1.1. Detailed parameters for the defined constants are listed below:

**Parameter**	**value**
Glucose diffusion coefficient	6.7×10^{-10} $m^2.s^{-1}$
Temperature	25 ˚C
Viscosity of water	0.69×10^{-3} Pa.s
Boltzmann constant	1.38×10^{-23} $J.K^{-1}$
Initial glucose concentration at the point source	1 $mol.L^{-1}$
The diameter of the circular domain	3.5 cm

Simulation code:

```
import numpy as np
import matplotlib.pyplot as plt
import math
from matplotlib.colors import LogNorm
from mpl_toolkits.mplot3d import Axes3D

# Set up parameters
D = 6.7e-10  # glucose diffusion coefficient in m^2/s
T = 25 + 273.15  # temperature in K
eta = 0.69e-3  # viscosity of water in Pa s
kB = 1.38e-23  # Boltzmann constant in J/K
alpha = (kB * T) / (6 * math.pi * eta * D)  # alpha parameter for diffusion
```

```
dt = 0.1  # time step in s
r = 0.0175  # radius of the circular domain in m
dx = dy = 0.0005  # spatial step size in m
nx, ny = int(2 * r / dx), int(round(2 * r / dy)) + 1  # number of grid points
C = np.zeros((nx, ny))  # concentration matrix

# Initialize point source at the center top point of the circular domain
source_x = int(nx / 2)
source_y = ny - 1
C[source_x, source_y] = 1

# Perform simulations at different times
times = [1200, 2400, 3600]  # simulation times in s
vmax = 1
vmin = 1e-50
for idx, t_final in enumerate(times):
    # Perform simulation
    for t in range(1, int(t_final / dt)):
        # Calculate second derivatives
        d2Cdx2 = np.zeros((nx, ny))
        d2Cdy2 = np.zeros((nx, ny))
        for i in range(1, nx - 1):
            for j in range(1, ny - 1):
                d2Cdx2[i, j] = (C[i + 1, j] - 2 * C[i, j] + C[i - 1, j]) / dx ** 2
                d2Cdy2[i, j] = (C[i, j + 1] - 2 * C[i, j] + C[i, j - 1]) / dy ** 2

# Update concentration matrix
        C[1:-1, 1:-1] += alpha * (d2Cdx2[1:-1, 1:-1] + d2Cdy2[1:-1, 1:-1]) * dt

        # Apply reflective boundary conditions
        C[0, :] = C[1, :]
        C[-1, :] = C[-2, :]
        C[:, 0] = C[:, 1]
        C[:, -1] = C[:, -2]
```

Supplementary Note S7. Notes about the chemotaxis of glucose powered nanorobots

In characterizing the behavior of CeSAN-bots, we describe their movement using the term "chemotaxis," albeit with a crucial distinction from the biological mechanisms. Unlike biological systems, where orientation changes are typically achieved through modulation of tumbling frequencies via sensing and signaling processes, the self-propelled colloids exhibit a different approach. Over the past decade, researchers have discovered that micro/nanomotors can exhibit artificial chemotaxis[11,12]. The explanation of this behavior has been categorized into two mechanisms: Janus particle reorientation and modulated rotational diffusion.

Firstly, there is a focus on particles with Janus asymmetry, where the particle constantly reorients itself either in the direction or opposite to a chemical gradient. This reorientation is achieved through a phoretic torque generated by variations in the reaction rate across the misaligned catalytic cap of the particle[13,14]. Another perspective involves the dependence of the rotational diffusion times of the motors on the fuel concentration. As the fuel concentration increases, the rotational diffusion time decreases, allowing the motors to travel longer linear stretches before reorienting. This mechanism shares intrinsic similarities with the biased tumbling frequency observed in run-and-tumble chemotaxis found in bacteria[11,15].

To further explore this behavior in our system, we implemented an agar diffusion assay, following the methodology previously reported[11,15]. The directional motion of CeSAN-bots is transient and does not lead to long-term accumulation of the particles in the vicinity of the high-concentration region - this is an expected outcome, although the collective motion of the motors is initially biased towards the agar cylinder, they do not possess any internal mechanism to accumulate at the site; therefore, they diffuse away, potentially diffusing into regions with low fuel concentrations. Although CeSAN-bots lack the strict Janus asymmetry required for particle reorientation, our observations demonstrate a dependence of rotational diffusion times on fuel concentration. After encountering the local maximum concentration, the accelerated chemokinesis behavior of these active colloids is followed by rapid dispersion because of the maximal motility. They make spatial

and temporal changes in response to chemical gradients, most likely due to active diffusion changing the translational and rotational speed of diffusion[11,12,16].

Several recent studies have challenged the long-standing assumption regarding the indispensability of the Janus structure in enzyme-powered micro/nanomotors. This assumption is based on the notion that heterogeneous coating of catalytic moieties, whether enzymes or inorganic materials with analogous catalytic activities, can generate a driving force through asymmetric catalytic reactions[17-19]. Patiño et al. reported that the threshold for urease enzyme numbers is rather important to break symmetry and generate motion, rather than its heterogenous distribution[18].

Significantly, research conducted by Archer and Ebbens[17] and Sun et al.[19] has provided insights suggesting that colloidal motors relying on H_2O_2 catalysis, facilitated by enzymes such as catalase or Pt, may not require pronounced Janus asymmetry, contrary to prior beliefs. Archer et al. further proposed that even subtle variations in surface reactivity, rather than macroscopic Janus asymmetry, could sufficiently disrupt symmetry and induce motion in these motors[17].

The current study adds to this discourse by introducing a glucose-oxidase mimicking configuration with Au single atoms decorated on CeO_2 surfaces. The finding that these single atoms should be heterogeneously distributed on the surface of the active colloid of CeNPs, rather than incorporated into its crystalline structure (as observed in the case of Fe), aligns with Archer and Ebbens's proposition. It suggests that inducing an obvious asymmetrical catalytic distribution in metal catalyst-based propulsion may not be essential, but rather, heterogeneity in reactivity across each nominal surface is inherent, implying an intrinsic heterogeneity that cannot be avoided.

Supplementary Note S8. Detailed experimental procedures for *in vitro* experiments

<u>Isolation and cultivation of mesenchymal stem cells (MSCs)</u>

Inguinal fat pads were harvested, cut into small pieces, and digested for 50 minutes in collagenase I (Sigma-Aldrich, St. Louis, Missouri, USA) diluted in Hanks' Balanced Salt solution (HBSS, final concentration 1 $mg.ml^{-1}$) at 37 ˚C. The resulting cell suspension was twice washed in Dulbecco´s Modified Eagle Medium (DMEM, Sigma-Aldrich) containing 10% Fetal Bovine Serum (Gibco by Life Technologies, Carlsbad, California, USA), antibiotics (100 $U.ml^{-1}$ of penicillin, 100 $\mu g.ml^{-1}$ of streptomycin) and 10 mM HEPES buffer (referred as a complete DMEM) and centrifuged at 250 g for 8 minutes. The cells were seeded in 15 ml of complete DMEM in 75-cm^2 tissue culture flasks (Techno Plastic Products, Trasadingen, Switzerland) and cultured at 37 ˚C in a humidified atmosphere of 5% CO_2. After 48 hours of cultivation, non-adherent cells were washed out and the adherent cells were cultured for an additional 10 days with a regular exchange of the medium and passaging of the cells to maintain their optimal concentration. The cells were harvested by incubation with 0.5% trypsin solution (Sigma-Aldrich) for 5 minutes at 37 ˚C followed by gentle scraping. The resulting cell suspensions were evaluated in terms of their purity (absence of CD11b and CD45 markers and presence of CD44 and CD105) by flow cytometry and differentiation potential to adipocytes and osteoblasts (data not shown) and was used in *in vitro* or *in vivo* experiments.

<u>WST assay</u>

To determine the effect of CeNPs on MSCs, their metabolic activity was assessed by water-soluble tetrazolium-1 (WST-1) assay (Roche, Mannheim, Germany). To allow MSCs to adhere before the addition of NPs, 1.5 x 10^4 cells/well were cultured in 96-well tissue culture plate (Nunc, Roskilde, Denmark) in 90 µl of complete DMEM for 24 hours at 37 ˚C in a humidified atmosphere of 5% CO_2. After 24 hours, selected concentrations of CeNPs and Au-CeNPs (7, 15, 31, 62, and 125 $\mu g.ml^{-1}$) diluted in complete DMEM were added to wells, and MSCs were cultured with NPs in a

total volume of 120 µl of complete DMEM for additional 48 hours. Control MSCs cultured without NPs were also included. WST-1 reagent was added to each well (12 µl/120 µl of the medium), the plates were incubated for an additional 2 hours and the absorbance was measured using spectrophotometer Sunrise (Tecan, Männedorf, Switzerland) and analyzed by Kim 32 software (Schoeller Instruments, Prague, Czech Republic).

Labelling of MSCs with PHK67 fluorescent dye

MSCs were labelled with PKH67 Green Fluorescent Cell Linker Kit (Sigma-Aldrich) to monitor their fate after intravitreal administration. MSCs were labelled according to the manufacturer´s instructions with modifications introduced in the protocol to achieve optimal labelling for MSCs isolated from adipose tissue. A final concentration of 2 µM of PKH67 for 1×10^6 MSCs/ml and a 5-minute incubation were determined to be optimal conditions. The fluorescence intensity and homogeneity of the staining were evaluated by flow cytometry (LSRII cytometer, BD Biosciences, Franklin Lakes, New Jersey, USA) and fluorescent microscopy (Microscope Axioskop, Zeiss, Oberkochen, Germany).

Supplementary Figures

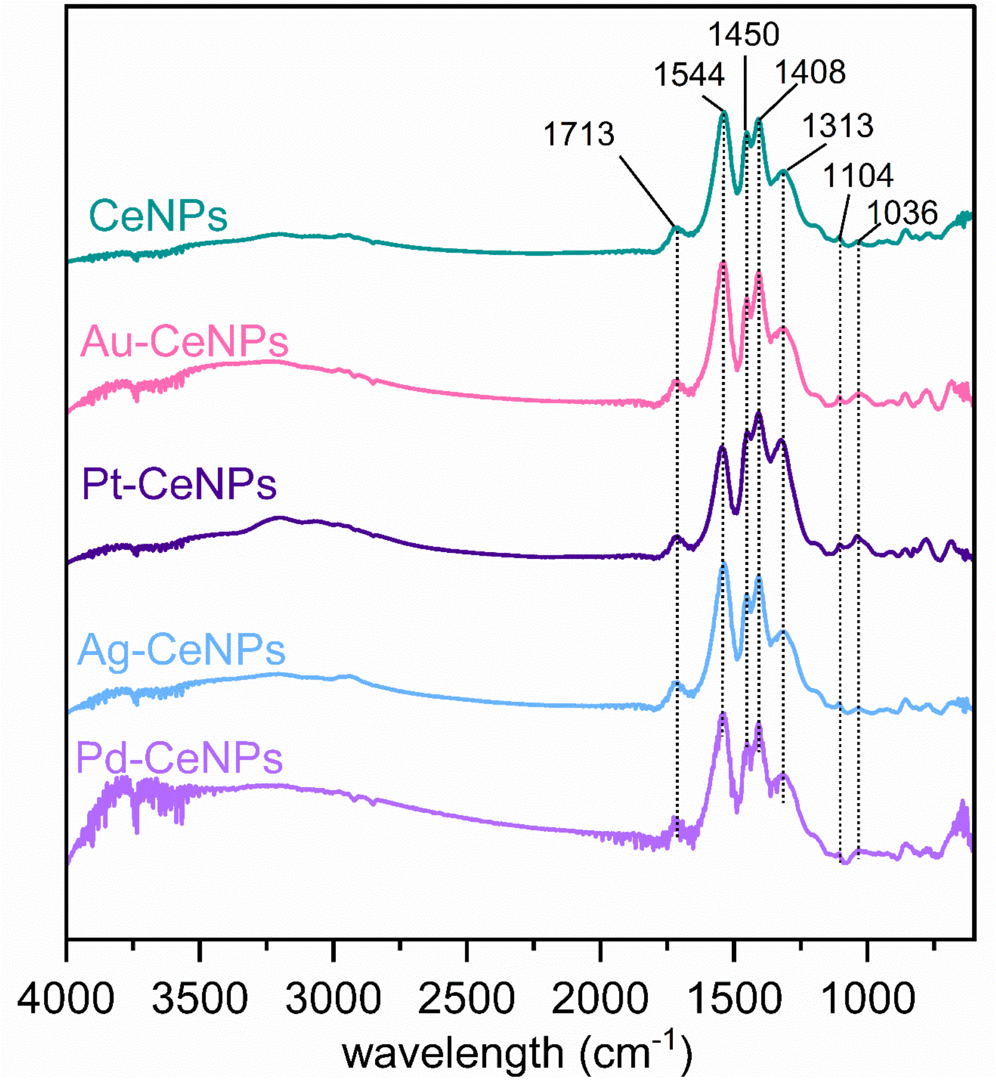

Supplementary Figure S1. ATR-FTIR characterization of poly(acrylic acid) coated nanoparticles. Selected individual peak assignments are as follows: 1) peak at 1713 cm^{-1} corresponds to C=O stretch; 2) 1544 cm^{-1} corresponds to $-COO^-$ asymmetric stretch; 3) peak at 1450 cm^{-1} corresponds to CH_2 or -C-OH bend; 4) peak at 1408 cm^{-1} corresponds to $-COO^-$ symmetric stretch. Peaks located from 1120 to 1000 cm^{-1} correspond to the C-OH stretch. These peak assignments have been previously reported[20] and correspond well to PAA-coated cerium oxide nanoparticles.

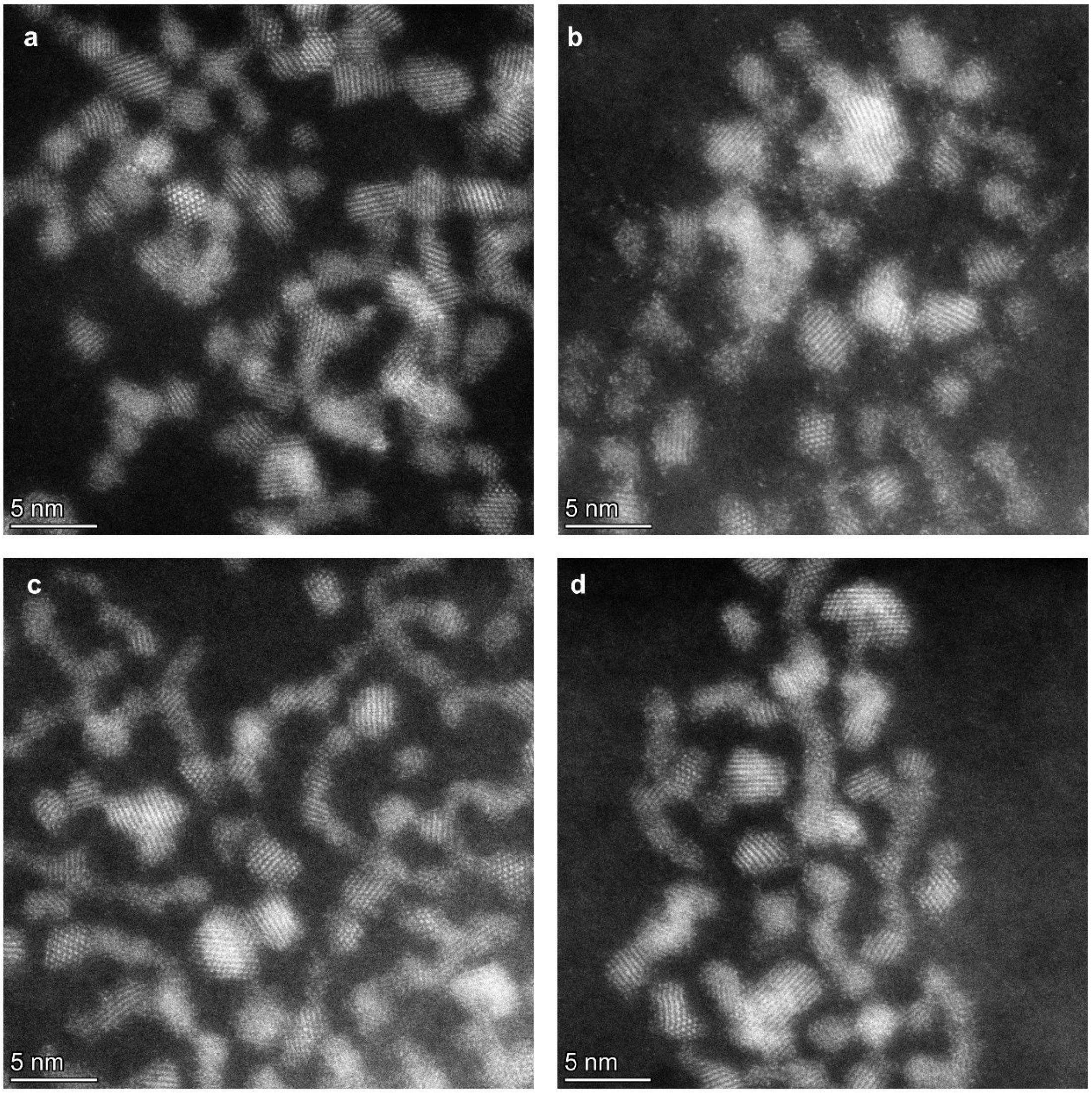

Supplementary Figure S2. HAADF-STEM images of CeSAN-bots a Au-CeNPs. **b** Pt-CeNPs. **c** Ag-CeNPs. **d** Pd-CeNPs.

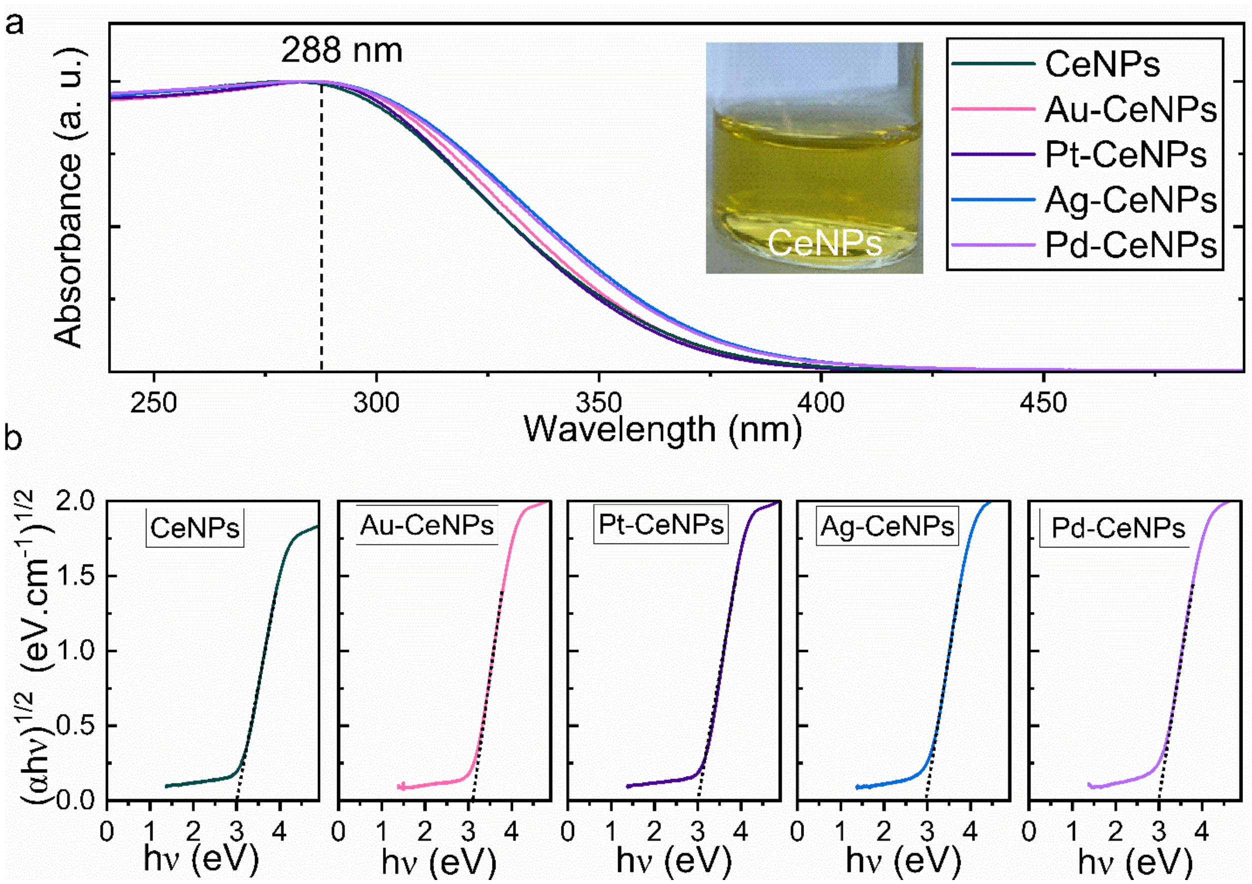

Supplementary Figure S3. UV-visible absorption spectra and bandgap calculation. a UV-visible absorbance spectra of CeNPs, Au-CeNPs, Pt-CeNPs, Ag-CeNPs, and Pd-CeNPs at diluted concentrations of 20 $mg.L^{-1}$. The displayed spectra were normalized based on the description in Supplementary Note S1. Picture of a vial with 20 $g.\ L^{-1}$ CeNPs dispersion is displayed as the insert. **b** The reflectance spectrum of CeNPs and M-CeNPs transformed according to the Tauc method[21]. The region shows a steep, linear increase in light absorption with increasing energy, which is characteristic of semiconducting materials. The linear part of the plot is extrapolated to the x-axis to determine the bandgap energy.

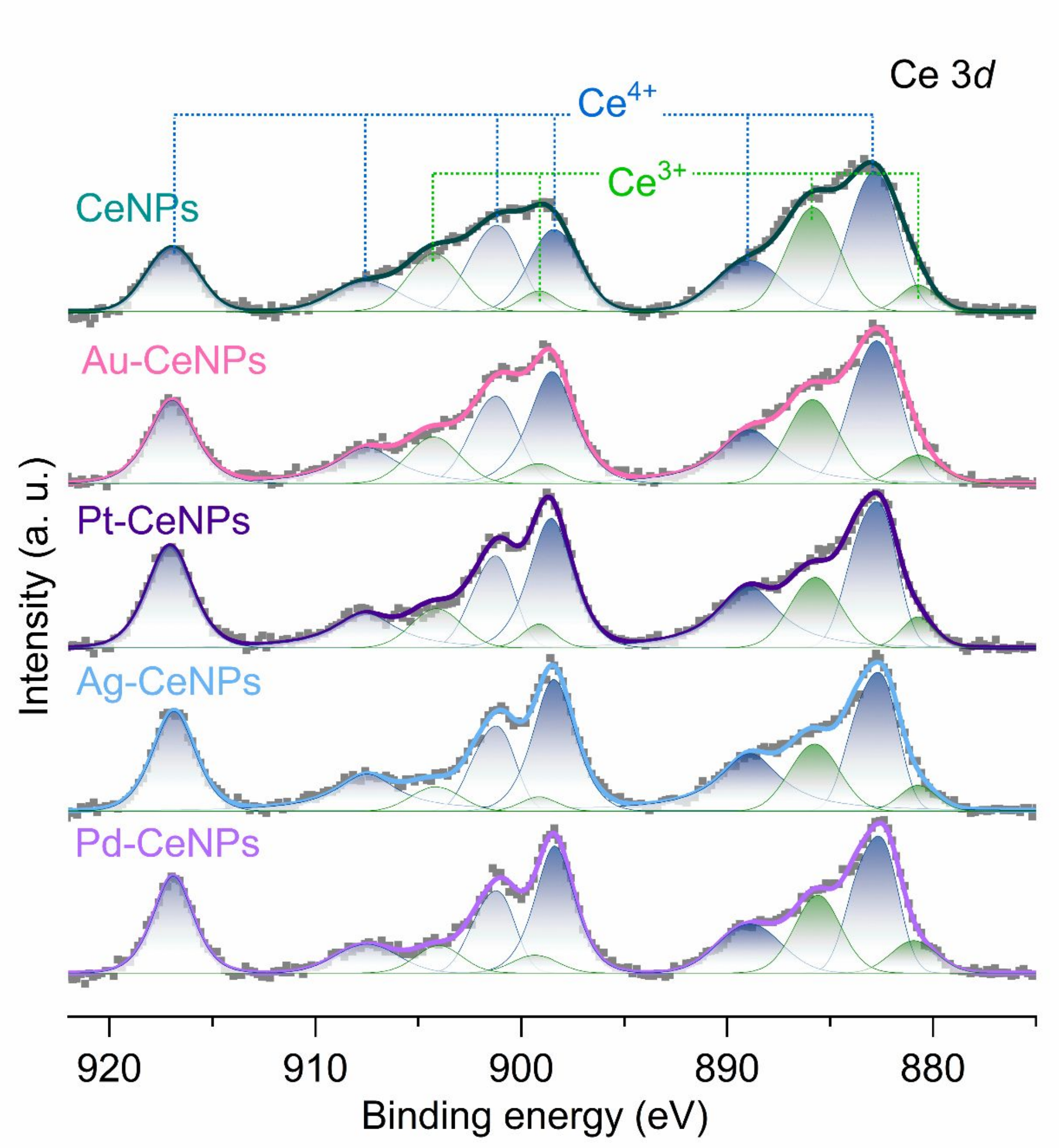

Supplementary Figure S4. XPS Ce 3*d* core-level spectra. XPS Ce 3*d* core-level spectra of synthesized CeNPs, Au-CeNPs, Pt-CeNPs, Ag-CeNPs, and Pd-CeNPs. There are three components associated with Ce^{4+} ions and two components associated with Ce^{3+} ions, and each of them is accompanied by their doublets of the Ce $3d_{5/2}$ and Ce $3d_{3/2}$ states, presenting a spin-orbit split of 18.5 eV. The fitting of the Ce^{3+} and Ce^{4+} peaks follows the parameters proposed by Lykhach et al[22]. Ranged Shirley backgrounds were subtracted, and doublet peaks were fitted by the Vogit doublet function using KolXPD software (kolibrik.net, s.r.o, Czech Republic). The Ce^{3+} fraction (Ce^{3+}%) was calculated from the integrated areas of the assigned peaks.

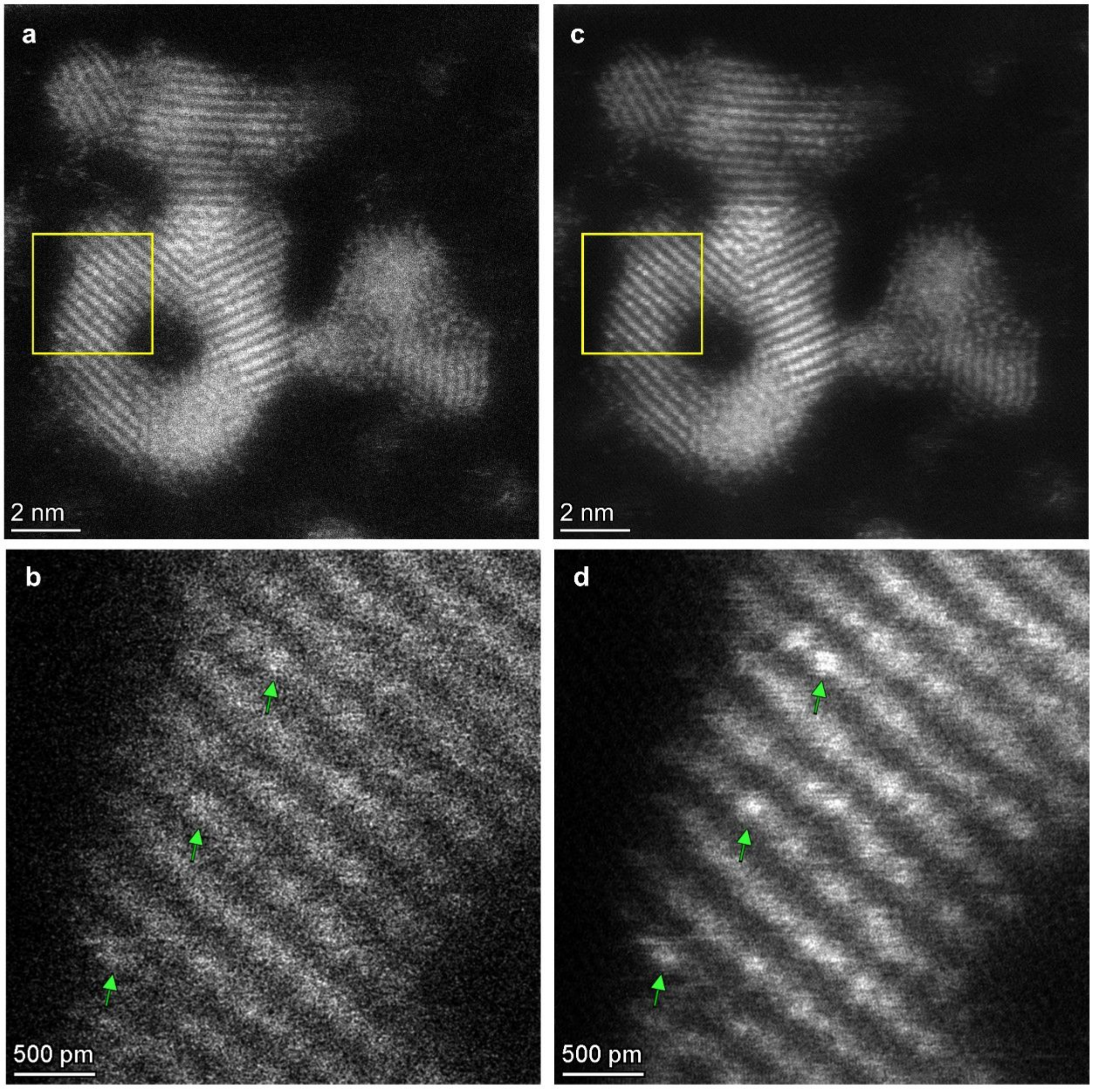

Supplementary Figure S5. High-resolution STEM images identifying Au single atoms on Au-CeNPs. a HAADF-STEM imaging condition with an increased collection angle range of 118 - 200 mrad, the yellow square is enlarged and shown in **b**. **b** Enlarged HAADF image, green arrows indicate single Au atoms. **c** MAADF-STEM (middle-angle ADF-STEM) imaging condition with 29 - 111 mrad collection angle range, yellow square is enlarged and shown in **c** as the same area as in **a**. **d** Enlarged MAADF image, green arrows with the same position as in **b** indicate single Au atoms with lower noise than in **b**. The increased signal intensity of single Au atom positions observed and indicated in **b, c** correlates, which approves the suitability of the used MAADF imaging condition to reveal Au atoms via the Z-contrast.

a

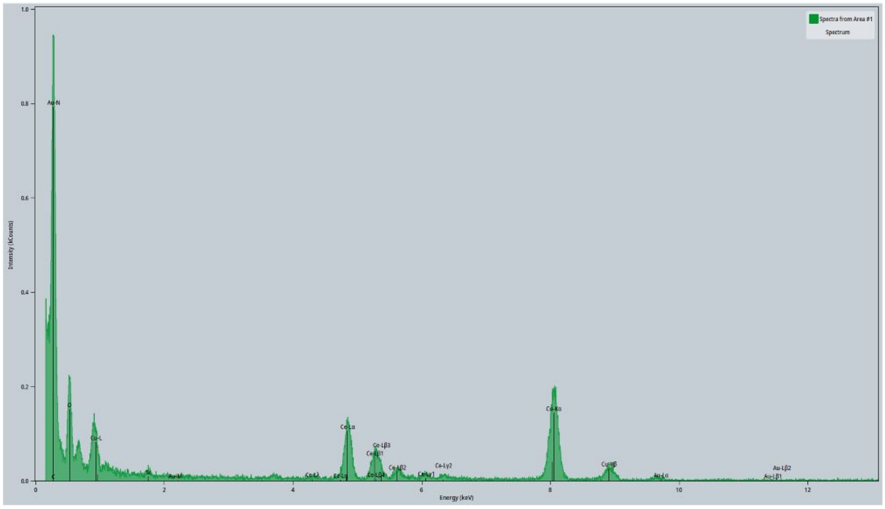

b

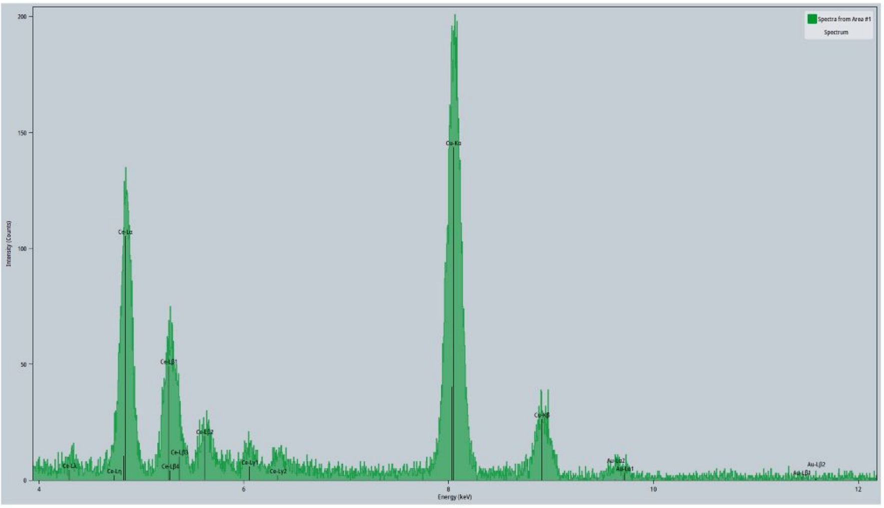

c

Z	Element	Family	Atomic Fraction (%)	Atomic Error (%)	Fit Error (%)
**8**	O	K	65.68	2.35	1.43
**58**	Ce	L	32.8	2.4	0.39
**79**	Au	L	1.51	0.18	5.87

Supplementary Figure S6. EDX spectra and elemental composition of Au-CeNPs. a Entire EDXS and **b** detailed spectra from the same area as shown in Figure 2g**. c** Table of O-K, Ce-L, and Au-L elemental quantification from the EDX spectra.

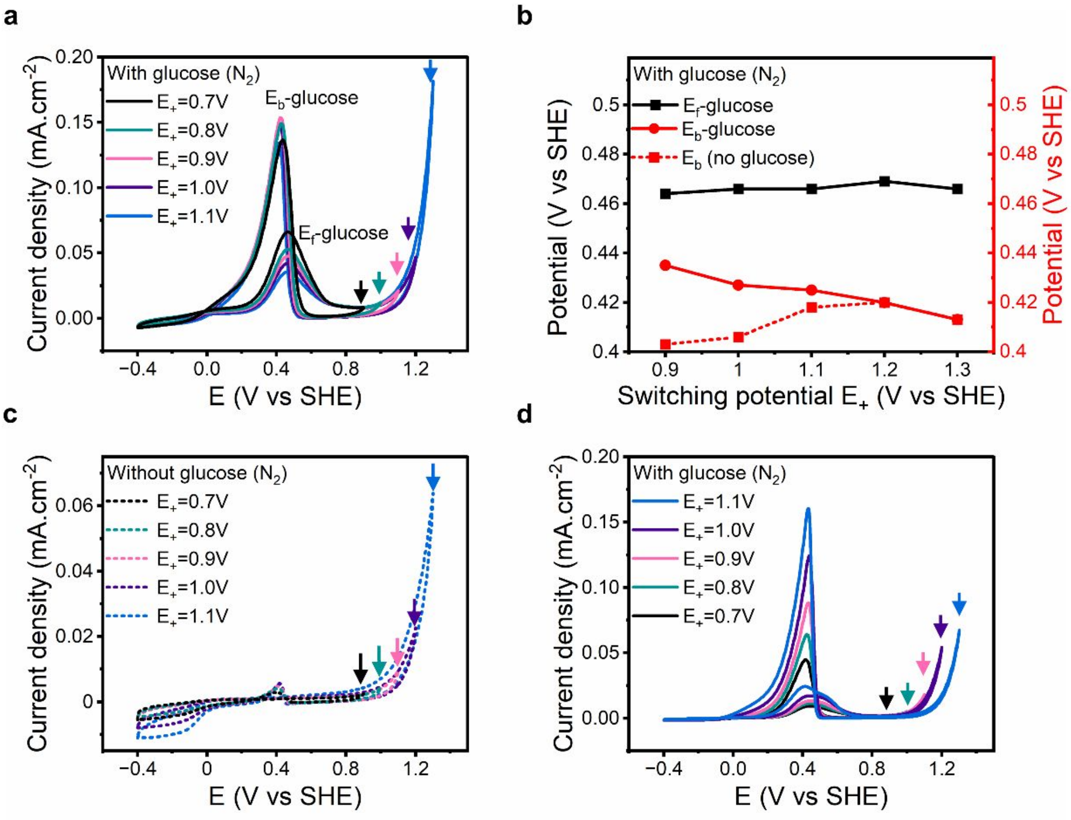

Supplementary Figure S7. The switching potential effect on glucose oxidation. a Effect of the oxidation extent of the Au-CeNPs electrode on E_f-glucose and E_b-glucose with glucose in the N_2-purged electrolyte, by tuning the switching potential in anodic scans from low voltage (E_+=0.7 V) to high voltage (E_+=1.1 V). **b** Dependence of various factors including E_f-glucose, E_b-glucose, and E_b (no glucose) on the switching potential E_+. **c** Switching potential effect of the oxidation extent of the Au-CeNPs electrode without glucose in the N_2-purged electrolyte, by tunning the switching potential in anodic scans from low voltage (E_+=0.7 V) to high voltage (E_+=1.1 V). **d** Effect of the oxidation extent of the Au-CeNPs electrode on E_f-glucose and E_b-glucose, by tuning the switching potential in anodic scans from high voltage (E_+=1.1 V) to low voltage (E_+=0.7 V), with 0.1V as step changes, with glucose in the N_2-purged electrolyte.

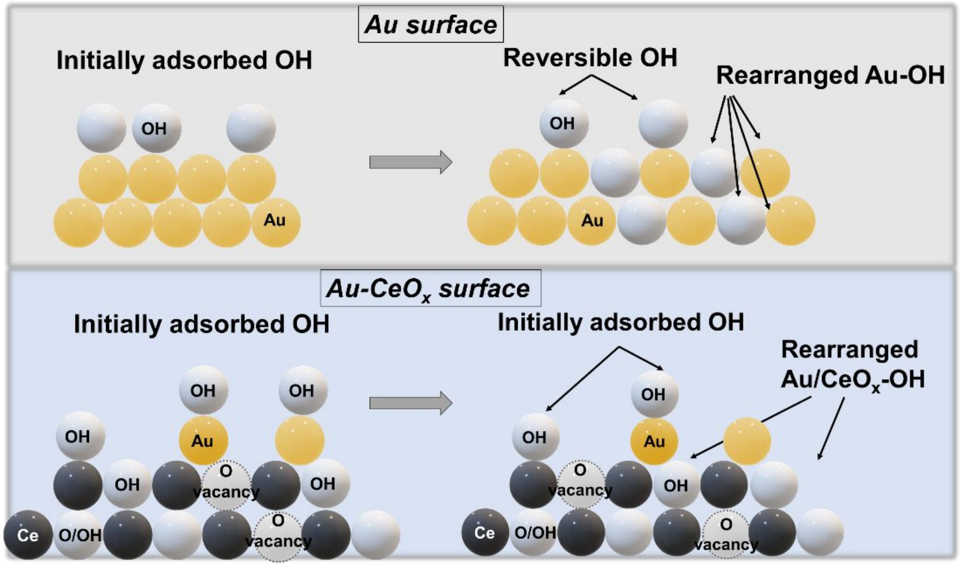

Supplementary Figure S8. Scheme of possible adsorption species on the Au-CeO_2 surface. Schematic diagrams of the increasing extent of deposition of OH species on an Au (upper panel) or Au-CeO_2 (lower panel) electrode surface with progressive transformation of an initially adsorbed OH to a place-exchanged Au-OH state. Color coding of atoms: white O/OH, grey Ce, golden Au.

a b c

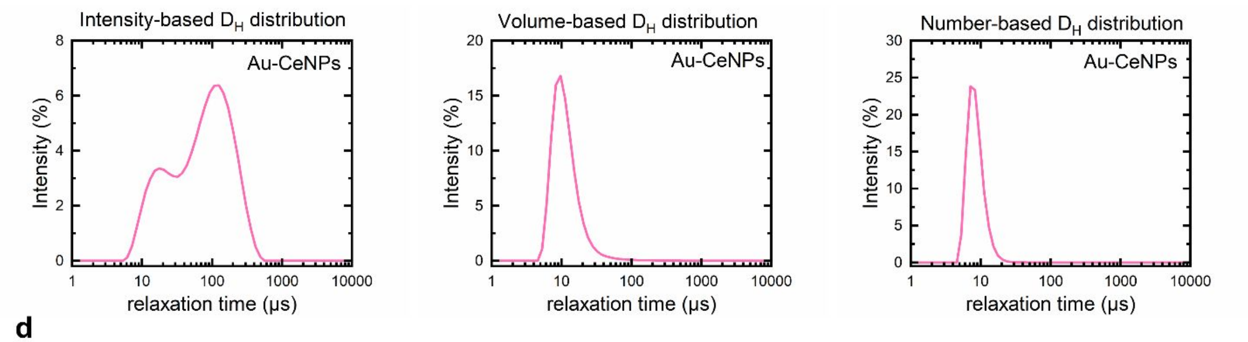

d

Name	D_H in PBS (nm) (number-based)	D_H in water (nm) (number-based)	Z-average (nm) (intensity-based)
CeNPs	5.2 ± 0.2	5.8 ± 0.2	32.7 ± 0.7
Au-CeNPs	9.1 ± 1.3	9.0 ± 3.2	40.2 ± 5.8
Pt-CeNPs	6.6 ± 0.6	7.3 ± 0.3	15.1 ± 7.3
Ag-CeNPs	53.0 ± 4.9	24.1 ± 5.8	108.4 ± 19.3
Pd-CeNPs	62.7 ± 6.4	23.6 ± 0.3	101.6 ± 9.8

Supplementary Figure S9. Hydrodynamic diameter by DLS. a-c Hydrodynamic diameter based on intensity (**a**), volume (**b**), and number (**c**) for Au-CeNPs. **d** Table of the colloidal hydrodynamic diameter (D_H, nm) of the synthesized CeNPs and M-CeNPs. The NPs were dispersed in distilled water and phosphate buffer saline (PBS) with a pH of 7.4. Diameter values are expressed on intensity-based values and number-based values. The intensity-based Z-average value (nm) of measured sizes is shown as Z-average.

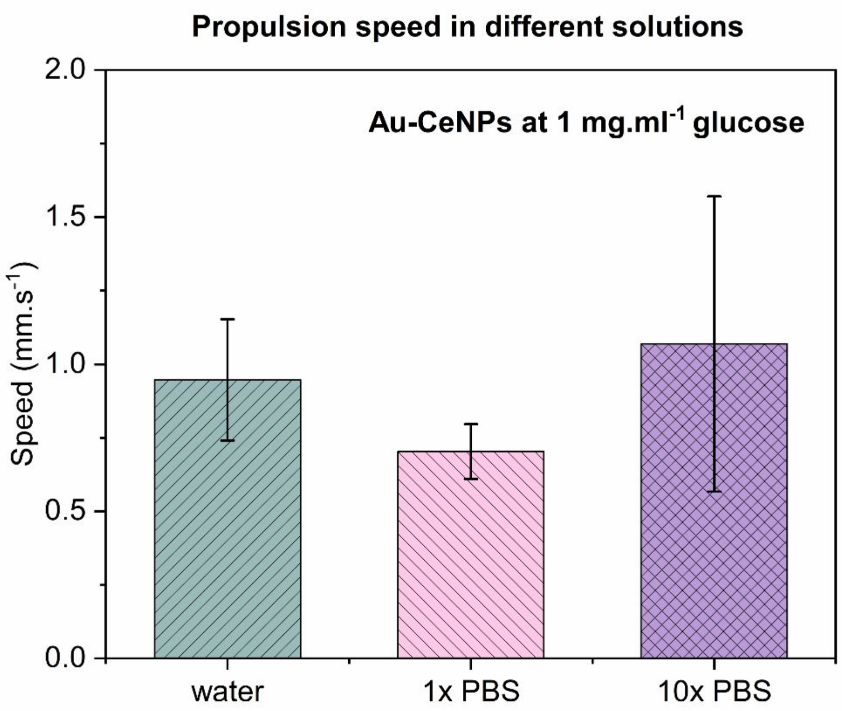

Supplementary Figure S10. Propulsion speed in different ionic strength solutions. Comparison of the average speed of Au-CeNPs based CeSAN-bots in solutions with different ionic strengths. The glucose concentration is 1 mg.ml^{-1}, equivalent to normal human blood sugar levels. There is no statistical significance between these three samples.

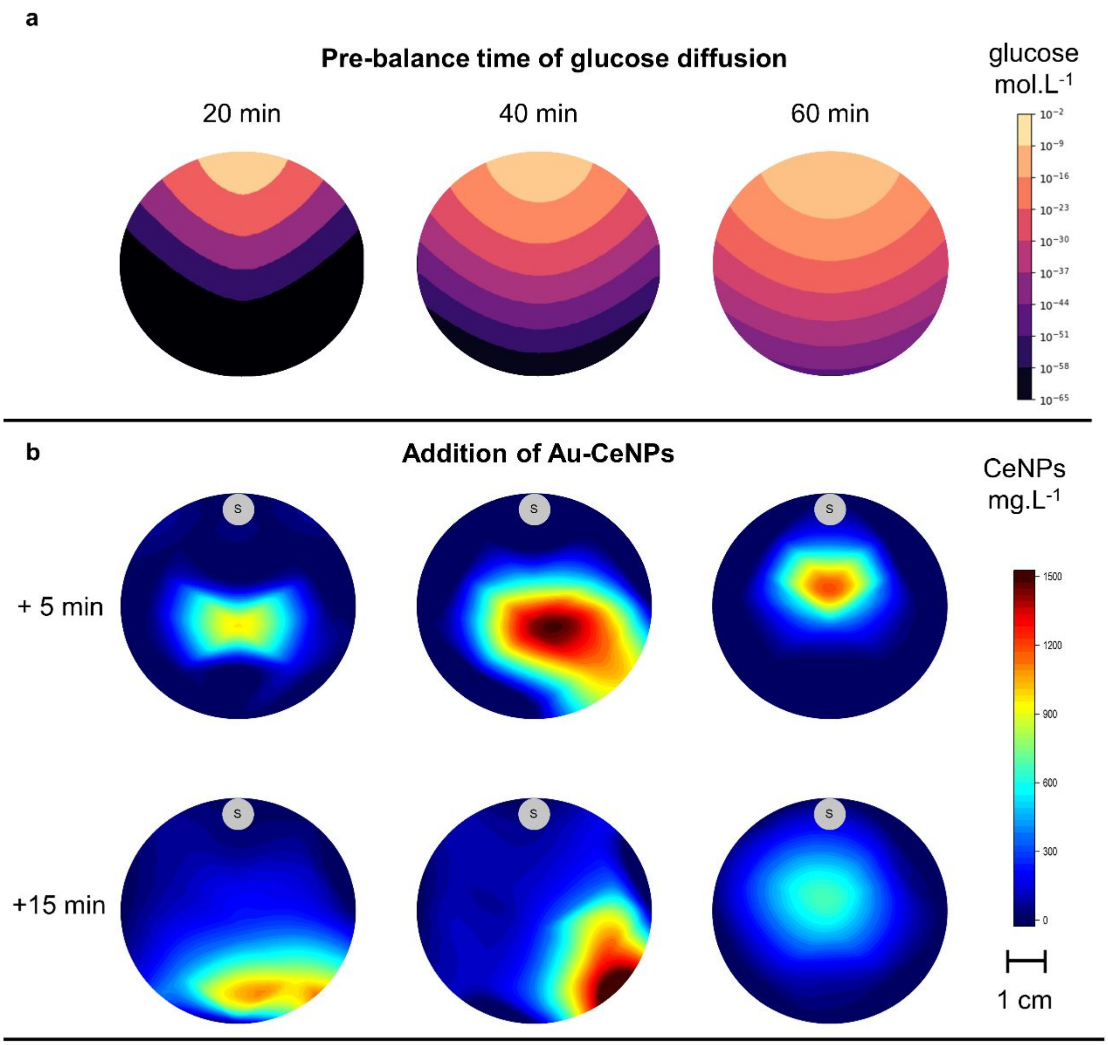

Supplementary Figure S11. Glucose diffusion and the effect of pre-balance time. a Simulated glucose diffusion concentration map depending on different pre-balance time for 20 minutes, 40 minutes, and 60 minutes. Simulation is carried out by Python and detailed code is presented in Supplementary Note S7. **b** Experimental data of CeNPs heatmap showing that the pre-balance time of glucose diffusion significantly influences the NPs distribution. For glucose pre-balance time of 20 and 40 minutes, after Au-CeNPs addition, the glucose diffusion dominates the Au-CeNPs diffusion, exhibiting a drift carried by the glucose diffusion toward the far edge away from the glucose source. While in the case of a pre-balance time of 60 minutes, the glucose drift is significantly minimized and the CeNPs distribution can be unbiasedly monitored. Thus, in all the data presented in the main text, a pre-balance time of 60 minutes of glucose diffusion is used for monitoring the CeNPs concentration due to diffusion/chemotaxis.

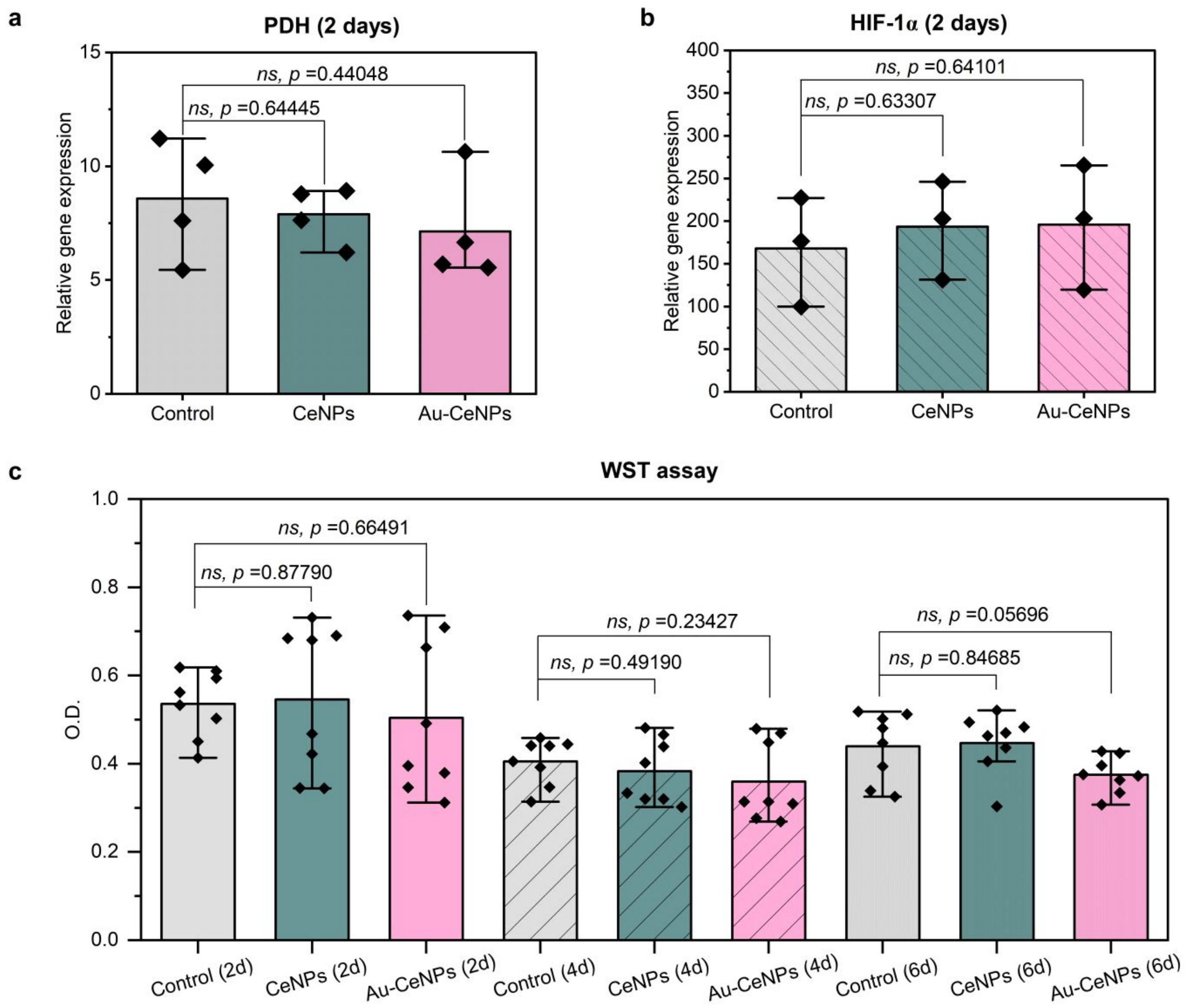

Supplementary Figure S12. The metabolism of MSCs cultured with CeSAN-bots. a Pyruvate dehydrogenase (PDH) gene regulation after 2 days of NPs incubation with MSCs. PDH gene encodes an enzyme complex that converts pyruvate into acetyl-CoA, linking glycolysis to the tricarboxylic acid (TCA) cycle, and its activity is tightly regulated by phosphorylation, nutrient availability, and metabolic signals to balance energy production and metabolic flux. **b** Hypoxia-inducible factor 1-alpha (HIF-1α) regulation after 2 days of NPs incubation with MSCs. HIF-1α is a transcription factor that regulates cellular adaptation to low oxygen conditions by promoting glycolysis, angiogenesis, and metabolic reprogramming, while suppressing mitochondrial oxidative metabolism through the inhibition of PDH. **c** Metabolic activity of MSCs co-incubated with CeSAN-bots tested with 2-day intervals up to 6 days.

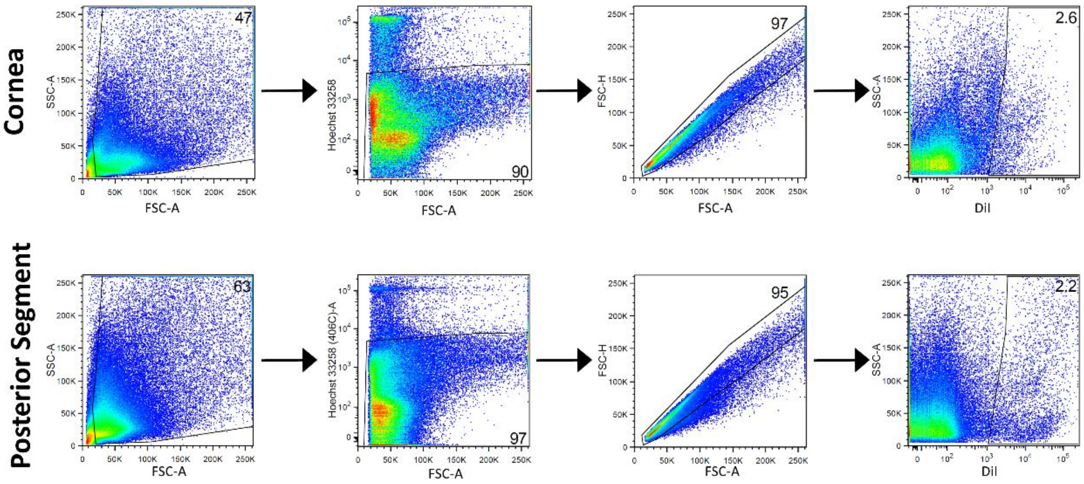

Supplementary Figure S13: General flow cytometry gating strategy for detection of DiI$^+$ cells in cornea (upper panel) and posterior segment (lower panel). Series of dot plots showing sequential gating of cellular events, live cells, single cells and DiI$^+$ cells.

Supplementary Table S1. Comparison of glucose-powered nanorobots: material design and performance metrics.

Engine	***Vector***	***Mechanism***	***Performance metrics***	***Size***
GOx+bilirubin oxidase[23]	Carbon fiber at water-air interface	Bioelectrochemical reaction	1 cm.s^{-1} for 20 s, stopped after 3 mins	0.5-1 cm
GOx+catalase[24]	PEG-Au-SAM fluid pump	Fluid density gradient	1.2 µm.s^{-1} at 50 mM glucose	N/A
GOx+catalase[25]	Stomatocyte	Enzyme-driven	10 µm.s^{-1} at 10mM glucose	~500 nm
GOx+catalase[15]	Polymersome	Chemotactic external gradient	200 µm.s^{-1} at 1M glucose	~50 nm
GOx+catalase[26]	ZIF-8	Buoyancy driven	60 µm.s^{-1} at 13 mM glucose	~800 nm
GOx+catalase[27]	Mesoporous organosilica	Self-diffusiophoresis	6.49 µm.s^{-1} at 200 mM glucose	~350 nm
GOx+Catalase[28]	Dendritic mesoporous silica	Self-diffusiophoresis	Chemotactic velocity of 1.48 µm.s^{-1} at 20 mM glucose	~300 nm
GOx[29]	Janus hollow mesoporous silica NPs	Self-diffusiophoretic force	24% increase at 75 mM glucose	~400 nm
GOx[30]	Janus magnetic FeO_x NPs	Self-diffusiophoresis	23% increase at 60 mM glucose	~200 nm
GOx[31]	Silica microcapsules	Self-diffusiophoresis	~0.1 µm.s^{-1}	~ 2 µm
Cu_2O microsphere[32]	N-CNTs	Photocatalytic diffusiophoresis	18.71 µm.s^{-1} at 30 mM glucose + light	~1.5 µm
AuNPs[33]	Pt hollow nest	Self-diffusiophoresis	20.4 µm.s^{-1} at 50 mM glucose	~60 nm
AuNPs[34]	Mesoporous silica NPs	Nitric acid bubble propulsion	10 µm.s^{-1} at 55 mM glucose	~80 nm
Au single atoms (current study)	Cerium oxide	Self-diffusiophoresis	Apparent velocity at 0.7 mm.s^{-1}; ~80% increase of diffusion coefficient; chemotactic velocity of 25 µm.s^{-1} at 55 mM glucose	~10 nm

Supplementary Table S2. Comparison of chemically-powered nanorobots for intracellular delivery.

Nanomotor type	Fuel	Application	Key findings
Cr/Pt-mesoporous silica Janus motors[35]	H_2O_2	Drug delivery to HeLa cells (DOX)	Effective delivery to HeLa cells, sub-100 nm
Paramagnetic/Pt microrobots[36]	H_2O_2	Targeted drug delivery	Practical but limited by fuel concentration
PtNPs-loaded hybrid stomatocyte nanomotors[37]	H_2O_2	Controlled intracellular DOX delivery	Autonomous motion under low fuel concentration, efficient drug delivery
Biocatalytic porous framework micromotors[38]	H_2O_2	Drug delivery to cancer cells	Reversible motility at low fuel concentration, pH responsive drug delivery
Urease-powered nanomotors[39]	Urea	DOX delivery to HeLa cells	Improved delivery efficiency compared to passive NPs
Au-Pt egg-in-nest nanomotor[33]	Glucose	Intracellular DOX delivery	efficient cellular uptake, pH induced drug release

Supplementary Table S3: Murine primer sequences used for real-time PCR

Gene	Sense primer	Antisense primer
**GAPDH**	AGAACATCATCCCTGCATCC	ACATTGGGGGTAGGAACAC
**p53**	GTATTTCACCCTCAAGATCC	TGGGCATCCTTTAACTCTA
**NGF**	TGGACTGCACGACCACAG	AAATTAGGCTCCCTGGAGGT
**PD-L1**	CTACGGTGGTGCGGACTACA	CATGCTCAGAAGTGGCTGCAT
**IL-6**	GCTACCAAACTGGATATAATCAGGA	CCAGGTAGCTATGGTACTCCAGAA
**K12**	GTGAGTCCGCTGGTGGTAAC	CATCAGCACAGCAGGAAGTG
**IGF-1**	TCGGCCTCATAGTACCCACT	ACGACATGATGTGTATCTTTATTGC
**Bax**	GTGAGCGGCTGCTTGTCT	GGTCCCGAAGTAGGAGAGGA
**Bcl-2**	AGTACCTGAACCGGCATCTG	GGGGCCATATAGTTCCACAAA
**PDH**	TGTGACCTTCATCGGCTAGAA	TGATCCGCCTTTAGCTCCATC
**HIF-1α**	AACTTCTGGATGCCGGTGG	TCGCCGTCATCTGTTAGCAC

References

1 Lopés-Velasco, N. M. & Bailón-Ruiz, S. J. Effect of the particle size and pH on the photocatalytic performance of cerium oxide (CeO_2) nanoparticles. *MRS Advances* **6**, 769-773 (2021).

2 Qi, L. *et al.* Redispersible hybrid nanopowders: cerium oxide nanoparticle complexes with phosphonated-PEG oligomers. *ACS Nano* **2**, 879-888 (2008).

3 Truffault, L. *et al.* Application of nanostructured Ca doped CeO_2 for ultraviolet filtration. *Materials Research Bulletin* **45**, 527-535 (2010).

4 Zhang, H., Liang, X., Han, L. & Li, F. "Non-naked" gold with glucose oxidase-like activity: a nanozyme for tandem catalysis. *Small* **14**, 1803256 (2018).

5 Chen, J. *et al.* Glucose-oxidase like catalytic mechanism of noble metal nanozymes. *Nature Communications* **12**, 3375 (2021).

6 Hwang, D.-W., Lee, S., Seo, M. & Chung, T. D. Recent advances in electrochemical non-enzymatic glucose sensors – A review. *Analytica Chimica Acta* **1033**, 1-34 (2018).

7 Hsiao, M. W., Adžić, R. R. & Yeager, E. B. Electrochemical oxidation of glucose on single crystal and polycrystalline gold surfaces in phosphate buffer. *Journal of The Electrochemical Society* **143**, 759 (1996).

8 Vassilyev, Y. B., Khazova, O. A. & Nikolaeva, N. N. Kinetics and mechanism of glucose electrooxidation on different electrode-catalysts: Part I. Adsorption and oxidation on platinum. *Journal of Electroanalytical Chemistry and Interfacial Electrochemistry* **196**, 105-125 (1985).

9 Conway, B. E. Electrochemical oxide film formation at noble metals as a surface-chemical process. *Progress in Surface Science* **49**, 331-452 (1995).

10 Lee, T.-C. *et al.* Self-propelling nanomotors in the presence of strong Brownian forces. *Nano Letters* **14**, 2407-2412 (2014).

11 Hong, Y., Blackman, N. M. K., Kopp, N. D., Sen, A. & Velegol, D. Chemotaxis of nonbiological colloidal rods. *Physical Review Letters* **99**, 178103 (2007).

12 Popescu, M. N., Uspal, W. E., Bechinger, C. & Fischer, P. Chemotaxis of active Janus nanoparticles. *Nano Letters* **18**, 5345-5349 (2018).

13 Xiao, Z., Nsamela, A., Garlan, B. & Simmchen, J. A platform for stop-flow gradient generation to investigate chemotaxis. *Angewandte Chemie International Edition* **61**, e202117768 (2022).

14 Mou, F. *et al.* ZnO-based micromotors fueled by CO_2: the first example of self-reorientation-induced biomimetic chemotaxis. *National Science Review* **8** (2021).

15 Joseph, A. *et al.* Chemotactic synthetic vesicles: design and applications in blood-brain barrier crossing. *Science Advances* **3** (2017).

16 Moran, J. L., Wheat, P. M., Marine, N. A. & Posner, J. D. Chemokinesis-driven accumulation of active colloids in low-mobility regions of fuel gradients. *Scientific Reports* **11**, 4785 (2021).

17 Archer, R. J. & Ebbens, S. J. Symmetrical catalytic colloids display Janus-like active brownian particle motion. *Advanced Science* **10**, 2303154 (2023).
18 Patiño, T. *et al.* Influence of enzyme quantity and distribution on the self-propulsion of non-Janus urease-powered micromotors. *Journal of the American Chemical Society* **140**, 7896-7903 (2018).
19 Sun, J., Wu, J. & Ju, H. Effects of size and asymmetry on catalase-powered silica micro/nanomotors. *Chemistry – An Asian Journal* **19**, e202300900 (2024).
20 Ju, X. *et al.* Poly(acrylic acid)-mediated synthesis of cerium oxide nanoparticles with variable oxidation states and their effect on regulating the intracellular ROS level. *Journal of Materials Chemistry B* **9**, 8530-8530 (2021).
21 Makuła, P., Pacia, M. & Macyk, W. How to correctly determine the band gap energy of modified demiconductor photocatalysts based on UV–vis spectra. *The Journal of Physical Chemistry Letters* **9**, 6814-6817 (2018).
22 Lykhach, Y. *et al.* Counting electrons on supported nanoparticles. *Nature Materials* **15**, 284-288 (2016).
23 Mano, N. & Heller, A. Bioelectrochemical propulsion. *Journal of the American Chemical Society* **127**, 11574-11575 (2005).
24 Sengupta, S. *et al.* Self-powered enzyme micropumps. *Nature Chemistry* **6**, 415-422 (2014).
25 Abdelmohsen, L. K. E. A. *et al.* Dynamic loading and unloading of proteins in polymeric stomatocytes: formation of an enzyme-loaded supramolecular nanomotor. *ACS Nano* **10**, 2652-2660 (2016).
26 Guo, Z. *et al.* Self-propelled initiative collision at microelectrodes with vertically mobile micromotors. *Angewandte Chemie International Edition* **61**, e202209747 (2022).
27 Liu, M. L. *et al.* Enzyme-based mesoporous nanomotors with near-Infrared optical brakes. *Journal of the American Chemical Society* **144**, 3892-3901 (2022).
28 Lin, J. *et al.* Hyperglycemia Targeting Nanomotors for Accelerated Healing of Diabetic Wounds by Efficient Microenvironment Remodeling. *Advanced Functional Materials* **n/a**, 2417146.
29 Ma, X. *et al.* Enzyme-powered hollow mesoporous Janus nanomotors. *Nano Letters* **15**, 7043-7050 (2015).
30 Rucinskaite, G., Thompson, S. A., Paterson, S. & de la Rica, R. Enzyme-coated Janus nanoparticles that selectively bind cell receptors as a function of the concentration of glucose. *Nanoscale* **9**, 5404-5407 (2017).
31 Arqué, X. *et al.* Intrinsic enzymatic properties modulate the self-propulsion of micromotors. *Nature Communications* **10**, 2826 (2019).
32 Wang, Q. *et al.* Glucose-Fueled Micromotors with Highly Efficient Visible-Light Photocatalytic Propulsion. *ACS Applied Materials & Interfaces* **11**, 6201-6207 (2019).
33 Kwon, T. *et al.* Au/Pt-Egg-in-Nest Nanomotor for Glucose-Powered Catalytic Motion and Enhanced Molecular Transport to Living Cells. *Angewandte Chemie International Edition* **60**, 17579-17586 (2021).

34 Zheng, J. *et al.* Cascade Catalytically Released Nitric Oxide-Driven Nanomotor with Enhanced Penetration for Antibiofilm. *Small* **18**, 2205252 (2022).
35 Xuan, M., Shao, J., Lin, X., Dai, L. & He, Q. Self-Propelled Janus Mesoporous Silica Nanomotors with Sub-100 nm Diameters for Drug Encapsulation and Delivery. *Chemphyschem* **15**, 2255-2260 (2014).
36 Villa, K. *et al.* Cooperative Multifunctional Self-Propelled Paramagnetic Microrobots with Chemical Handles for Cell Manipulation and Drug Delivery. *Advanced Functional Materials* **28**, 1804343 (2018).
37 Tu, Y. *et al.* Biodegradable Hybrid Stomatocyte Nanomotors for Drug Delivery. *ACS Nano* **11**, 1957-1963 (2017).
38 Gao, S. *et al.* Superassembled Biocatalytic Porous Framework Micromotors with Reversible and Sensitive pH-Speed Regulation at Ultralow Physiological H_2O_2 Concentration. *Advanced Functional Materials* **29**, 1808900 (2019).
39 Hortelão, A. C., Patiño, T., Perez-Jiménez, A., Blanco, À. & Sánchez, S. Enzyme-Powered Nanobots Enhance Anticancer Drug Delivery. *Advanced Functional Materials* **28**, 1705086 (2018).
